# Noninvasive Mechanical Ventilation Improves Breathing-Swallowing Interaction of Ventilator Dependent Neuromuscular Patients: A Prospective Crossover Study

**DOI:** 10.1371/journal.pone.0148673

**Published:** 2016-03-03

**Authors:** Marine Garguilo, Michèle Lejaille, Isabelle Vaugier, David Orlikowski, Nicolas Terzi, Frédéric Lofaso, Hélène Prigent

**Affiliations:** 1 UMR1179, University of Versailles Saint-Quentin-en-Yvelines, Versailles, France; 2 CIC INSERM 1429 - Raymond Poincaré Hospital, AP-HP, Garches, France; 3 Home ventilation unit (Intensive Care Department), Raymond Poincaré Hospital, AP-HP, Garches, France; 4 Intensive Care Department, CHRU Caen, Caen, France; 5 INSERM U1075 - Université de Caen, Caen, France; 6 Physiology Department, Raymond Poincaré Hospital, AP-HP, Garches, France; Erasmus Medical Centre, NETHERLANDS

## Abstract

**Background:**

Respiratory involvement in neuromuscular disorders may contribute to impaired breathing-swallowing interactions, swallowing disorders and malnutrition. We investigated whether the use of non-invasive ventilation (NIV) controlled by the patient could improve swallowing performances in a population of neuromuscular patients requiring daytime NIV.

**Methods:**

Ten neuromuscular patients with severe respiratory failure requiring extensive NIV use were studied while swallowing without and with NIV (while ventilated with a modified ventilator allowing the patient to withhold ventilation as desired). Breathing-swallowing interactions were investigated by chin electromyography, cervical piezoelectric sensor, nasal flow recording and inductive plethysmography. Two water-bolus sizes (5 and 10ml) and a textured yogurt bolus were tested in a random order.

**Results:**

NIV use significantly improved swallowing fragmentation (defined as the number of respiratory interruption of the swallowing of a single bolus) (p = 0.003) and breathing-swallowing synchronization (with a significant increase of swallows followed by an expiration) (p <0.0001). Patient exhibited piecemeal swallowing which was not influenced by NIV use (p = 0.07). NIV use also significantly reduced dyspnea during swallowing (p = 0.04) while preserving swallowing comfort, regardless of bolus type.

**Conclusion:**

The use of patient controlled NIV improves swallowing parameters in patients with severe neuromuscular respiratory failure requiring daytime NIV, without impairing swallowing comfort.

**Trial Registration:**

ClinicalTrials.gov NCT01519388

## Introduction

The increasing use of non-invasive mechanical ventilation (NIV) associated with the increasing ventilator-dependence of neuromuscular patients with progressive respiratory failure has led some patients to extend NIV use during daytime [1, 2].

At that stage of neuromuscular disorder (NMD) with respiratory muscle dysfunction, swallowing disorders due to deficiency of upper airway muscles are also frequently observed [3, 4] and can lead to malnutrition [5, 6]. These swallowing disorders may also be worsened by the respiratory failure. Indeed, while in healthy adults swallows are predominantly followed by expiration [7–9], patients with respiratory insufficiency due to either chronic obstructive pulmonary disease (COPD) [10, 11] or to NMD [12, 13] equally swallow during the expiratory and inspiratory phases of respiration. Expiration-followed swallow is a mechanism helpful in clearing the pharyngeal recesses of foreign residues before subsequent inspiration and may prevent low-grade recurrent aspiration. Moreover, when swallowing interrupts the expiratory phase of the respiratory cycle, the elastic recoil of the lungs and the chest wall can generate a subglottic positive pressure which is considered as a key component of swallowing efficiency [14–17].

Recently Terzi et al. [18] demonstrated that relieving the urgency to breathe during acute respiratory failure in COPD patients by using nasal mechanical ventilation improved swallowing performances. To avoid inadequate ventilator insufflations, they used a prototype, developed from an existing ventilator (Elysée 150, ResMed Corp, Sans Diego, USA) and modified by its manufacturer in order to be controlled by the patient through a contactor which when activated by the patient withholds air delivery by the ventilator.

As we observed that neuromuscular patients with severe chronic respiratory muscle dysfunction also present breathing/swallowing desynchronisation [12, 13] as COPD patients with acute respiratory failure, we decided to evaluate whether the use of this mechanical ventilation device improved the breathing-swallowing interaction of neuromuscular patients with severe respiratory failure.

## Methods

### Study population

**Fig 1:**
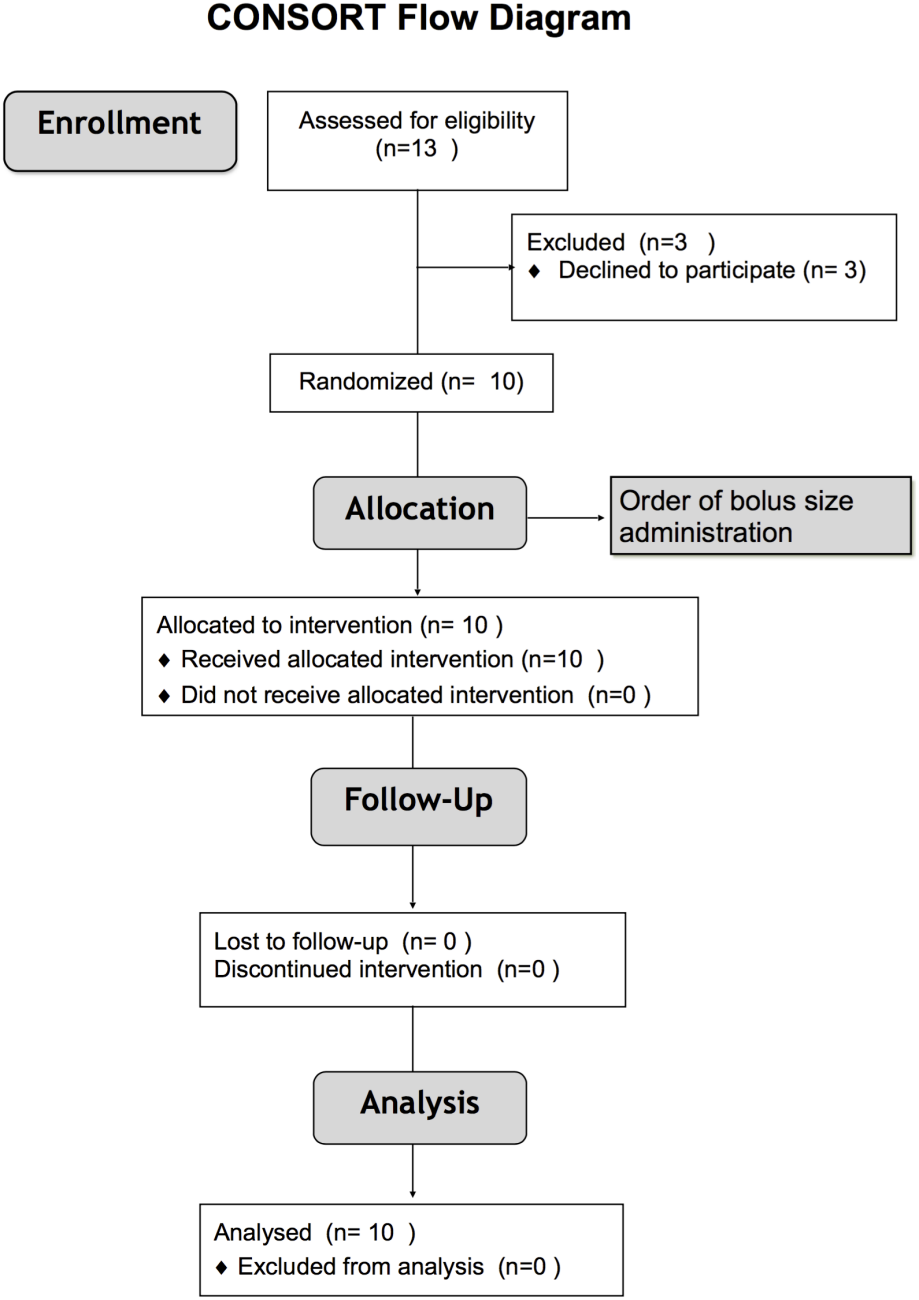
CONSORT study flow diagram.

From February 2012 until May 2013, we conducted a prospective, open-label, interventional, crossover study (fig 1) in the home mechanical ventilation unit of the Raymond Poincare University Hospital (Garches, France).

Adult stable patients with restrictive neuromuscular respiratory failure requiring extensive NIV (≥ 14h/day) were included during their usual respiratory follow-up. Patients with a gastrostomy were excluded. The patient’s usual NIV interface was used. The sample size was based on a cross-over design to test NIV versus spontaneous breathing (SB). If patients used both a nasal interface and a mouthpiece during eating, their usual nasal mask was used. As breathing-swallowing interactions are altered in the neuromuscular patients with an inverse correlation between respiratory muscles strength and breathing-swallowing dysfunction [12], the impact of the percentage of inspiration-followed swallows was chosen as primary endpoint. Considering that the number of swallows followed by an inspiration is estimated at 45% with spontaneous ventilation with a standard deviation of 25% in patients with severe respiratory failure [18] the enrolment of 10 patients will give a 80% power to detect a difference of 25% between NIV and SB assuming a standard error of 25% for this difference.

The appropriate ethics committee (CPP Ile de France XI, Ref 11074) approved the study. All patients provided written informed consent prepared by and addressed to the Clinical Investigation Center of our Hospital (CIC) labelled by the French Minister of Health. The protocol was registered in clinical trial (ClinicalTrials.gov NCT01519388).

### Experimental setup

Thoracic and abdominal movements were monitored using respiratory inductive plethysmography (Respitrace; Ambulatory Monitoring, Ardsley, NY, USA). Flow was measured using a pneumotachograph (Fleisch#2, Switzerland). Swallowing was monitored noninvasively using electromyography (EMG) to detect submental muscle activity via skin-surface electrodes on the chin and a piezoelectric sensor placed between the cricoid and thyroid cartilages on the midline to detect laryngeal motion [12, 19]. All signals were recorded on a computer equipped with the MP 150 data-acquisition system (Biopac Systems, Santa Barbara, CA, USA).

### Description of the prototype ventilator

The ventilator was a commercialized life support ventilator able to provide volumetric and assisted modes of ventilation (Elysée 150, ResMed SAS, ResMed Corp, Sans Diego, USA). It was altered with the help of its manufacturer, ResMed SAS (ResMed Corp, Sans Diego, USA), to allow interruption of ventilatory support during swallowing. A key-pinch off-switch was added to allow ventilation deactivation for as long as pressure was applied to the switch. The switch (Microleger 7C02, Ablenet Inc, Roseville, Mn, USA) was easily accessible by the patient and chosen for its ability to be used by patients with severely impaired motor function [20]. Thus, the patient was able to stop insufflations at will, for as long as he maintained pressure with his finger on the switch. Releasing the switch resulted in the immediate start of a new controlled cycle.

The patient’s usual mechanical ventilation settings were applied to the prototype ventilator and each patient was given a period of quiet breathing under NIV to adjust, if necessary, the ventilator settings.

### Experimental protocol

Participation lasted for less than 3 hours. Each patient was seated comfortably, and the experiment was started after a period of quiet breathing. The head was maintained in the neutral position to avoid bias due to effects of position on swallowing [21]. Water boluses were placed in the mouth using a syringe. Five sets of two boluses sizes were used (5 and 10 mL) and were studied in random order. The patients were blinded to bolus size. They were instructed to swallow normally while trying to be as efficient as possible. In the absence of aspiration with water, patients were recorded while swallowing the sequence of five teaspoons (5ml) of textured flavored yogurt (Danette, Danone^®^, Paris, France), in order to reproduce swallowing close to natural situation, with a texture type usually recommended for patients with swallowing disorders. Swallowing was tested with and without NIV in random order using only one block size generated by independent statistician from the CIC and sealed in opaque envelopes.

### Data analysis

The recording examiner (MG) was blinded from pressure and flow signals in order to be also blinded to the presence or not of nasal mechanical ventilation. Swallowing onset was defined as the onset of phasic submental EMG activity and swallowing termination as the beginning of the downward laryngeal movement detected by the piezoelectric sensor [19]. For each bolus size, duration of swallowing, number of swallows, and fragmentation of swallowing by respiratory events (defined as the number of respiratory interruptions (inspiration, expiration or both) of a single bolus swallowing) were recorded. Using respiratory movement direction (inspiration or expiration) immediately after each swallow, the percentage of swallows followed by inspiration (the sequence believed to contribute to aspiration during swallowing [11, 22]) were computed for each patient. Dyspnea while swallowing was assessed with the modified Borg scale (from 0 = “No Dyspnea” to 10 = “Maximal Dyspnea”) at the end of each swallowing trial. Likewise, swallowing comfort was evaluated with a visual analog scale (VAS) (from 0 = extremely uncomfortable to 10 = extremely comfortable). The percentage of swallows followed by an inspiration was considered as the primary outcome whereas other parameters were considered as secondary outcomes. Data were monitored by the CIC, which is independent from the investigators and the sponsors. They were anonymized and kept in the database which was password-protected, was authorized by the CNIL (French Data Protection Authority) and available only to the authors.

All results are reported as means ± standard deviations in the text and as means ± standard error of the mean in figures. Statistical tests were run using the StatView 5 package (SAS Institute, Grenoble, France). Two-way analysis of variance with repeated measures (NIV effect, bolus-texture effect, bolus-size effect) was performed. Statistical significance was declared at the 0.05 level.

## Results

### Study population

During the inclusion period, ten neuromuscular ventilator-dependent patients, whose characteristics are shown in Table 1, were studied. Mean age was 33.0±15.2 years. All patients had severe neuromuscular respiratory failure (vital capacity: 16.4±13.3% of predicted value) with ventilator dependency (17.7±3.9h per day). All patients were ventilated and presented an important restriction of mouth opening with Mallampati score of 3 or above.

**Table 1:** Patients characteristics.

Patient	Sex	Diagnostic	Age (years)	BMI (kg/m²)	VC (L) (% of predicted)	NIV duration (h/day)	UsualMeal duration (min)	Mallampati score (/4)
1	M	DMD	26	17.6	0.50 (11)	14	50	4
2	M	DMD	20	23.7	0.54 (14)	20	120	4
3	M	DMD	20	16.0	0.41 (9)	21	60	4
4	F	LGMD	44	20.7	0.53 (17)	22	20	3
5	M	DMD	24	9.5	0.30 (8)	22	20	4
6	F	CM	44	23.0	1.84 (46)	23	NA	3
7	M	DMD	38	22.2	0.41 (8)	19	40	4
8	F	LGMD	67	26.2	0.85 (35)	14	45	3
9	M	DMD	23	13.7	0.38 (9)	14	60	3
10	M	DMD	24	11.2	0.34 (7)	14	30	4

BMI: body mass index; VC: Vital capacity, NIV: non-invasive ventilation; NA: not available; CM: congenital myasthenia, DMD: Duchenne muscular dystrophy; LGMD: limb girdle muscular dystrophy.

Usual mean meal duration was 50.0±30.5 min (Patient#6 was unable to provide an evaluation of her meal duration). Two patients (#5 and #7) were used to eating under NIV with their usual mask while two other patients (#1 and #4) only occasionally used it. All the other patients were not usually ventilated during meals

### Swallowing variables

**Fig 2:**
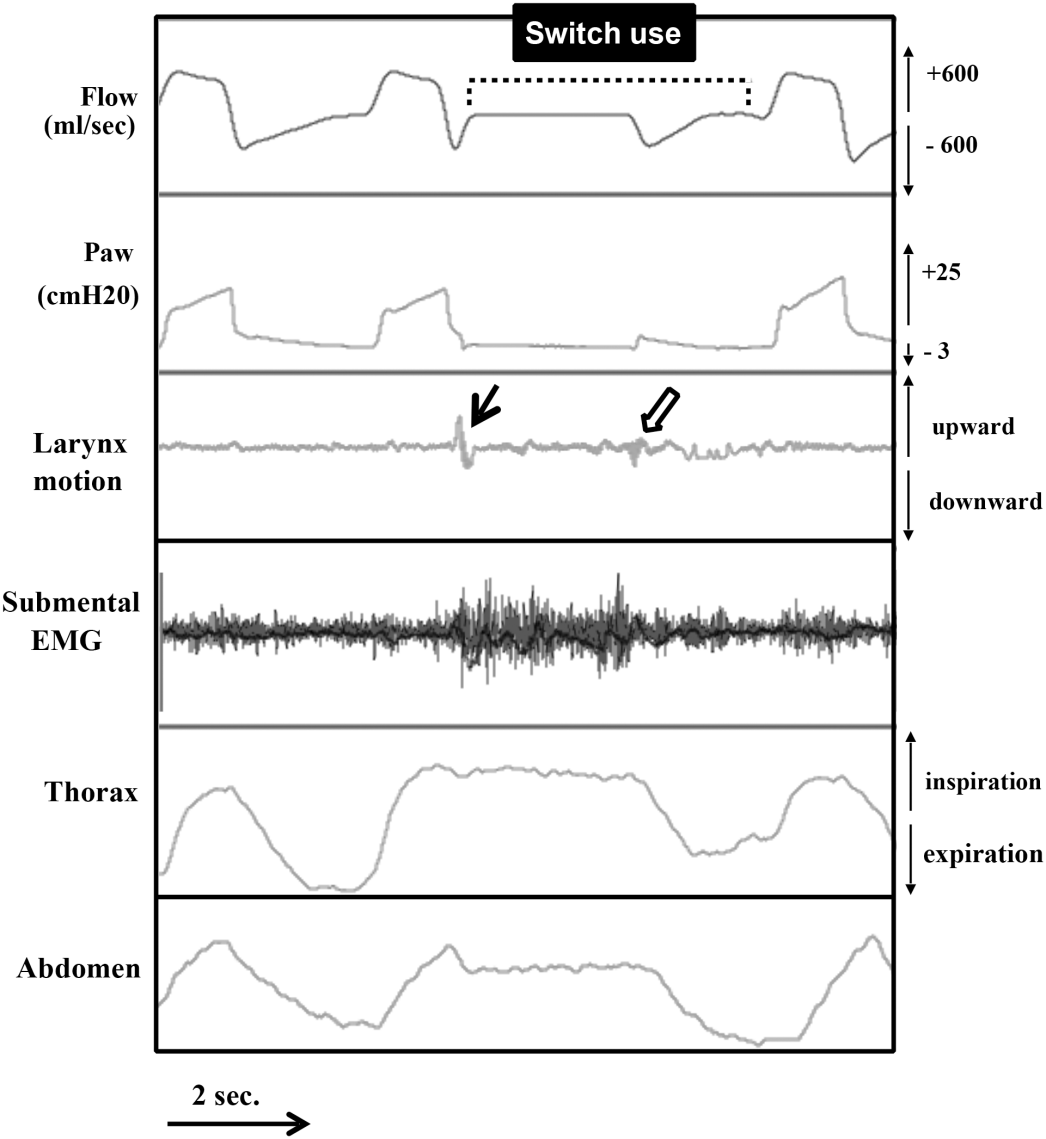
Switch use during swallowing under NIV. The patient withholds mechanical ventilation at the end of the inspiratory cycle by activating the switch and starts to swallow (plain arrow). The end of swallowing is followed by a reopening of the glottis which generates a positive pressure in the upper airways (shallow arrow) and by a spontaneous expiration of the patient as evidenced by the thoracic and abdominal movements. The patient then resumes his mechanical ventilation by deactivating the switch.

All the patients used the switch continuously during each swallow with NIV (therefore withholding mechanical ventilation throughout the swallow of each bolus) even if they resumed spontaneous breathing and fragmented swallowing (Fig 2).

Neither clinically significant aspiration nor coughing was observed during swallowing either during spontaneous breathing or during the use of NIV.

### Coordination between swallowing and respiration

The percentage of swallows followed by inspiration was not influenced by bolus size or texture and was above 40% in all cases during spontaneous breathing. It decreased significantly with NIV use with the bolus type (Table 2).

**Table 2:** Swallowing variables per bolus according to bolus type (5ml, 10ml, or yogurt) and test condition: during spontaneous breathing (SB) and with NIV.

	5 ml-bolus		10 ml-bolus		5ml-Yogourt		ANOVA (p)			
	SB	NIV	SB	NIV	SB	NIV	Interaction	Bolus size effect	Bolus texture effect	NIV effect
Number of swallows (per bolus)	2.0±0.9	2.2±1.1	2.8±1.1	2.9±1.4	2.6 ±1.2	2.4±1.1	0.39	<0.0001	0.89	0.98
Duration of swallowing (sec)	5.4±4.6	4.6±3.4	7.1±4.5	5.9±3.4	7.1±4.9	5.8±4.2	0.93	0.12	0.30	0.08
Swallowing fragmentation (respiratory events per bolus)	1.6±1.8	0.8±1.0	2.3±1.7	1.0±1.4	1.9±1.5	1.1±1.0	0.53	0.16	0.74	<0.0001
% of swallows followed by an inspiration	43.5±23.3	10.3±7.7	46.1±23.6	17.9±19.5	45.7±21.5	21.1 ±16.4	0.78	0.20	0.14	<0.0001
Borg Scale	2.4±2.2	1.2±1.6	2.7±2.2	1.1±1.5	2.9±2.4	1.7±1.5	0.89	0.80	0.36	0.0002

### Patients’ sensations

Dyspnea sensation during swallowing decreased significantly with NIV use for each bolus type (Table 2) while NIV use did not compromise swallowing comfort (VAS: 5ml-bolus: 6.9±2.2 in SB vs 7.3±1.4 during NIV, 10ml-bolus: 7.9±0.9 in SB vs 7.3±2.6 during NIV, yogurt-bolus: 6.9±2.4 in SB vs 7.3±2.8 during NIV (P = 0.46)).

When asked, all the patients considered that the device would be useful in their daily life.

## Discussion

The use of a patient-controlled NIV during swallowing significantly improved breathing-swallowing coordination in ventilated neuromuscular patients with severe respiratory failure by increasing the pattern of expiration-followed swallowing and reducing dyspnea. The studies of healthy adults have shown that the apnea during which swallowing occurs is predominantly followed by expiration [7–9]. This mechanism is considered to contribute to the safety of the airways during swallowing, as the expiratory flow may prevent the inhalation of remnants of the swallowed bolus when the glottis reopens after swallowing apnea [11, 23, 24]. This pattern is altered in patients with respiratory disease, both in COPD [10, 11] and in neuromuscular disorders [12, 13]. Accordingly, we observed that, during spontaneous breathing, our patients exhibited an impaired coordination between respiration and swallowing, as more than 40% of swallowing apneas were followed by an inspiration regardless of the type of bolus swallowed. Experimentally, such disruptions are reproduced in healthy subjects exposed to increased respiratory muscle elastic loading and to hypercapnia[22, 25]. Therefore, the correction of those factors by mechanical ventilation may lead to an improvement of breathing swallowing interactions. Accordingly, we observed in tracheostomized patients that the parameters of swallowing improved when patients swallowed while mechanically ventilated [12, 13, 22]. Therefore, unloading the respiratory muscles with mechanical ventilation is liable to improve breathing swallowing patterns. Moreover, positive pressure ventilation may also contribute to increase subglottic pressure during expiration by increasing the inspiratory volume which in turn increases the recoil pressure of the respiratory system [14, 16]. Low subglottic pressure may induce an increased duration of bolus transit and therefore increase the pharyngeal residue and the risk of aspiration [26, 27]. In this population with severe respiratory failure, the usual respiratory pattern during spontaneous breathing is rapid shallow breathing with low tidal volumes [28, 29]. Therefore, the increased tidal volume obtained with NIV is likely to participate in swallowing improvement.

With the improvement of interfaces and ventilators’ technology, NIV has been increasingly used throughout the day allowing the non-invasive management of severe patients, even in case of ventilator dependency, with an improved survival [2]. However, malnutrition and weight loss are frequently observed in that population, often imposing an invasive management through gastrostomy feeding [30]. Therefore, improving swallowing under NIV could participate in improving management and quality of life of neuromuscular patients, especially if it allows to avoid or to delay the use of such invasive methods [30, 31]. Even if malnutrition and gastrostomy may not be avoided with this technique, it may allow maintaining a hedonistic oral eating for specific favorite dishes while reducing unpleasant sensations such as dyspnea.

While the use of NIV could be perceived as an additional constraint, it did not impair swallowing parameters and even improved significantly swallowing fragmentation. Likewise, the comfort of swallowing was not significantly altered by the use of NIV. However, dyspnea during swallowing significantly decreased with NIV use. Patients felt an immediate benefit in terms of respiratory comfort and all of them found the device to be potentially useful in their daily life. It is interesting to note that some patients already used NIV during their meals, for the relief perceived despite the possible patient ventilator asynchrony that could be induced by swallowing [18].

We did not observe a reduction in the number of swallows per bolus contrary to what Terzi et al observed in COPD patients [18]. However, while respiratory failure mainly accounted for the impairment of breathing-swallowing interactions in COPD patients, swallowing impairment, in neuromuscular patients, may result from different mechanisms, as the underlying disease may alter swallowing muscles performances. In that situation, NIV may improve the dysfunction linked to respiratory failure but swallowing dysfunction linked to the neuromuscular disorder remains.

NIV has been increasingly administered during daytime through mouthpiece in ventilator dependent patients. It is not compatible during the entire process of chewing followed by swallowing. NIV use with a nasal mask, on the other hand, allows patients to chew while being ventilated and to choose when to interrupt ventilation in order to swallow which accounts for the improvement of the synchronization of swallow with the ventilatory cycle. Interestingly, among the four patients who spontaneously used NIV during meals, two had a mouthpiece but preferred to use their nasal mask for swallowing.

One limitation of the study was that our method of evaluation of breathing and swallowing interaction did not formally exclude silent microaspirations. Only invasive techniques, such as fiberoptic endoscopic exam [32–34], could ascertain the absence of such events but they are not compatible with a non-invasive ventilation of good quality, as their use would generate leaks impairing ventilation efficiency. We favored a method which placed the patients in a situation closest to their usual settings for swallowing (in their usual sitting position and installation…) with the least possible interactions with the ventilation and the swallowing performances.

The choice of switch is essential in order to optimize safety for these patients with majorly impaired motor function and who require appropriate interfaces to control their environment [20]. To ensure that, we used a very sensitive key-pinch switch (Microleger 7C02, Ablenet Inc, Roseville, Mn, USA) that only required 10g of strength to be activated.

As the evaluation of the swallowing improvement was only short-term, it is not possible to assert whether these improvements of breathing-swallowing interactions observed persist over time, especially in evolving NMD. Moreover, to ascertain whether they are sufficient to improve the widely observed malnutrition in the neuromuscular population [6, 35] would require to study its long term use and effectiveness at home, especially as malnutrition may also result from swallowing impairment linked to the underlying disease. Nevertheless, all the patients expressed their interest in the device and found it potentially useful for their daily life.

In conclusion, the use of a patient-controlled NIV during swallowing showed a significant improvement of breathing-swallowing coordination and therefore reducing the risk on breathing-swallowing dysfunction and of aspiration. NIV use was associated with an immediate reduction of dyspnea perceived during swallowing while maintaining the swallowing comfort. Whether these improvements allow to correct the malnutrition and weight loss commonly observed in this population and to avoid resorting to invasive feeding method needs to be evaluated prospectively in the home setting.

## Supporting Information

S1 CONSORT Checklist CONSORT checklist. (PDF)

S1 Protocol Trial Protocol. (PDF)

S2 Protocol Translated Trial Protocol. (PDF)
