# Supplementary material for: Noninvasive Mechanical Ventilation Improves Breathing-Swallowing Interaction of Ventilator Dependent Neuromuscular Patients: A Prospective Crossover Study
Source: PLoS One. 2016 Mar 3;11(3):e0148673. doi: 10.1371/journal.pone.0148673 (PMC4777441; doi:10.1371/journal.pone.0148673)
Supplement: S1 Protocol — (PDF) [file pone.0148673.s002.pdf]

ADEP ASSISTANCE

OptiDEG

Evaluation de l'efficacité de la ventilation nasale sur l'optimisation de la déglutition chez les sujets neuromusculaires ventilés

Etude pilote en cross-over, randomisée, en ouvert, versus ventilation spontanée.

Version n° 3 du 12/11/2012

Investigateur coordonnateur :
Dr Hélène Prigent
Physiologie Explorations Fonctionnelles, Centre d'Investigation Clinique et d'Innovation Technologique, Hôpital Raymond Poincaré, Garches.
Tel : 01 47 10 79 11   Fax : 01 47 10 79 43
Email :   helene.prigent@rpc.aphp.fr

Responsables scientifiques :
Principal : Pr Frédéric Lofaso
Physiologie Explorations Fonctionnelles, Centre d'Investigation Clinique et d'Innovation Technologique, Hôpital Raymond Poincaré, Garches.
Tel : 01 47 10 79 40   Fax : 01 47 10 79 43
Email : f.lofaso@rpc.aphp.fr
Associé : Dr. Nicolas Terzi
Service de réanimation médicale, CHU de Caen, Avenue de la Côte-de-Nacre,
14033 Caen Cedex, France
Tél : 02.31.06.52.68   Fax : 02.31.06.49.96
Email: terzi-n@chu-caen.fr

Investigateurs :
Dr David Orlikowski
Unité de Ventilation à domicile –Service de Réanimation médicale
Hôpital Raymond Poincaré, Garches
Tel : 01 47 10 77 76   Fax : 01 47 10 44 46
Email : david.orlikowski@rpc.aphp.fr

Equipe associée :
Orthophoniste
Marine Garguilo
Centre d'Investigation Clinique et d'Innovation Technologique
Physiologie Explorations Fonctionnelles,
Hôpital Raymond Poincaré, Garches.
Tel : 01 47 10 46 15 Fax : 01 47 10 79 33
Email : marine.garguilo@rpc.aphp.fr

Promoteur de l'étude :
ADEP assistance
Mme Annie Leroy
2 rue Benoît Melon 92150 Suresnes
Tel : 01 46 97 12 87 Fax : 01 46 97 16 94
E-mail : adepassistance@adep.asso.fr

Centre de Gestion et de Méthodologie :
Centre d'Investigation Clinique et d'Innovation Technologique (CIC-IT) :
Dr Frédéric Barbot
Hôpital Raymond Poincaré-104 avenue Raymond Poincaré-92380 Garches
Tél : 01.47.10.46.15 Fax : 01.47.10.46.33
Email : frederic.barbot@apr.aphp.fr

Page de SIGNATURE D'UN PROTOCOLE de recherche biomédicale par l'investigateur COORDONNATEUR et le représentant du PROMOTEUR

Recherche Biomédicale **N° ANSM : 2011-A00771-40**

Titre *:* **«Evaluation de l'efficacité de la ventilation nasale sur l'optimisation de la déglutition chez les sujets neuromusculaires ventilés**

Etude pilote en cross-over, randomisée, en ouvert, versus ventilation spontanée»

Version N° 3 du : 12/11/2012

L'investigateur coordonnateur :

Dr Hélène Prigent
Service de :
Physiologie Explorations Fonctionnelles, Centre d'Investigation Clinique et d'Innovation Technologique, Hôpital Raymond Poincaré, Garches
Hôpital Universitaire Raymond Poincaré
104 bd Raymond Poincaré – 92380 Garches

L'investigateur coordonnateur autorise également la diffusion de son identité et de ses coordonnées dans le répertoire des recherches biomédicales

Date : 07/11/2012

Signature :

Responsables scientifiques :

Pr Frédéric Lofaso
Physiologie Explorations Fonctionnelles, Centre d'Investigation Clinique et d'Innovation Technologique, Hôpital Raymond Poincaré, Garches.

Date : 12/11/2012

Signature :

Le promoteur :

ADEP assistance
Mme Annie Leroy
2 rue Benoît Melon 92150 Suresnes
Tel : 01 46 97 12 87 Fax : 01 46 97 16 94

Date : 14/12/2012

ADE[illegible]ISTANCE S.A.
2 [illegible]
[illegible]SURESNES
Tél. 01 46 97 12 87
Fax 01 46 97 16 94
Au capital de 12 500 000 €
SIREN : 487 778 151 RCS Paris

Signature :

1 RÉSUME DU PROJET. 6
2 JUSTIFICATION SCIENTIFIQUE ET DESCRIPTION GÉNÉRALE DE LA RECHERCHE. 7
2.1 DESCRIPTION GÉNÉRALE DU DISPOSITIF 7
2.2 RÉSUMÉ DES RÉSULTATS DES ESSAIS NON CLINIQUES ET CLINIQUES DISPONIBLES ET PERTINENTS 7
2.3 RÉSUMÉ DES BÉNÉFICES ET DES RISQUES PRÉVISIBLES ET CONNUS 7
2.4 DESCRIPTION DE LA POPULATION À ÉTUDIER. 7
3 OBJECTIFS DE LA RECHERCHE. 8
3.1 OBJECTIF PRINCIPAL 8
3.2 OBJECTIF(S) SECONDAIRE(S) 8
4 CONCEPTION DE LA RECHERCHE. 8
4.1 CRITÈRE DE JUGEMENT PRINCIPAL 8
4.2 CRITÈRE(S) DE JUGEMENT SECONDAIRE(S) 8
4.3 PLAN EXPÉRIMENTAL - DÉROULEMENT PRATIQUE 8
4.4 DESCRIPTION DES MESURES PRISES POUR RÉDUIRE ET ÉVITER LES BIAIS 9
4.5 DURÉE DE LA RECHERCHE 10
4.6 DESCRIPTION DES RÈGLES D'ARRÊT DÉFINITIF OU TEMPORAIRE 10
4.7 IDENTIFICATION DE TOUTES LES DONNÉES À RECUEILLIR DIRECTEMENT DANS LES CAHIERS D'OBSERVATION, QUI SERONT CONSIDÉRÉES COMME DES DONNÉES SOURCE. 10
5 SÉLECTION DES PERSONNES DE LA RECHERCHE 11
5.1 CRITÈRES D'INCLUSION 11
5.2 CRITÈRES DE NON-INCLUSION DES PERSONNES QUI SE PRÊTENT À LA RECHERCHE 11
5.3 PARTICIPATION SIMULTANÉE À UNE AUTRE RECHERCHE, PÉRIODE D'EXCLUSION 11
6 ARRÊT PRÉMATURÉ DE L'UTILISATION DU (DES) DISPOSITIF(S) 12
7 TRAITEMENT UTILISÉ AUTRE QUE LE DISPOSITIF FAISANT L'OBJET DE LA RECHERCHE 12
8 EVALUATION DE LA PERFORMANCE 12
9 EVALUATION DE LA SÉCURITÉ 12
9.1 DESCRIPTION DES PARAMÈTRES D'ÉVALUATION DE LA SÉCURITÉ 12
9.2 MÉTHODES ET CALENDRIER POUR MESURER, RECUEILLIR ET ANALYSER LES PARAMÈTRES D'ÉVALUATION DE LA SÉCURITÉ 13
9.3 PROCÉDURES MISES EN PLACE EN VUE DE L'ENREGISTREMENT ET DE LA NOTIFICATION DES ÉVÉNEMENTS INDÉSIRABLES 13
9.4. MODALITÉS ET DURÉE DU SUIVI DES PERSONNES SUITE À LA SURVENUE D'ÉVÈNEMENTS INDÉSIRABLES 15
10 STATISTIQUES.. 15
10.1 NOMBRE PRÉVU DE PERSONNES À INCLURE DANS LA RECHERCHE, ET NOMBRE PRÉVU DE PERSONNES DANS CHAQUE LIEU DE RECHERCHES AVEC SA JUSTIFICATION STATISTIQUE. 15
10.2 DESCRIPTION DES MÉTHODES STATISTIQUES PRÉVUES, Y COMPRIS DU CALENDRIER DES ANALYSES INTERMÉDIAIRES PRÉVUES. 15
10.3 MÉTHODES DE PRISE EN COMPTE DES DONNÉES MANQUANTES, INUTILISÉES OU NON VALIDES 16
10.4 ANALYSE INTERMÉDIAIRE 16

10.5 CHOIX DES PERSONNES À INCLURE DANS L'ANALYSE. 16
11 DROIT D'ACCÈS AUX DONNÉES ET DOCUMENTS SOURCE 16

12 CONTRÔLE ET ASSURANCE DE LA QUALITÉ 16
12.1 PROCÉDURES DE MONITORING 16
12.2 TRANSCRIPTION DES DONNÉES DANS LE CAHIER D'OBSERVATION 17
13 CONSIDÉRATIONS ÉTHIQUES.. 17
13.1 DEMANDE D'AUTORISATION AUPRÈS DE L'AUTORITÉ COMPÉTENTE 17
13.2 DEMANDE D'AVIS AU COMITÉ DE PROTECTION DES PERSONNES 17
13.3 DÉCLARATION CNIL 18
13.4 NOTE D'INFORMATION ET CONSENTEMENT ÉCLAIRÉ 18
13.5 RAPPORT FINAL DE LA RECHERCHE 18
14 TRAITEMENT DES DONNÉES ET ARCHIVAGE 18
15 FINANCEMENT ET ASSURANCE 19
15.1 ASSURANCE 19
15.2 ENGAGEMENT SCIENTIFIQUE 19
16 RÈGLES DE PUBLICATION 20
17 BIBLIOGRAPHIE 20

1 RÉSUME DU PROJET.

Contexte :

Les pathologies neuromusculaires peuvent être associées à des troubles de la déglutition du fait d'une déficience des muscles des voies aériennes impliquées dans l'acte de déglutition [1]. Nous avons démontré que l'insuffisance respiratoire pouvait également participer à ces anomalies de la déglutition, et que lorsque l'indication de la trachéotomie était posée, parce qu'elle permettait une ventilation mécanique au cours de la déglutition, elle améliorait la déglutition [2-3] malgré l'effet délétère communément reconnue de la trachéotomie seule sur la déglutition [4]. L'objectif est donc maintenant de démontrer que la ventilation non-invasive nasale pourrait être bénéfique sur la déglutition moyennant quelques adaptations permettant une bonne synchronisation entre la ventilation et la déglutition. Cela pourrait permettre d'éviter la trachéotomie ou la gastrostomie secondaires à des troubles de déglutition et/ou à une dénutrition. L'amélioration de la déglutition sous ventilation mécanique passe techniquement par l'absence de désynchronisation entre le ventilateur et le patient au moment de la déglutition. Sur ce point, une première étude a débuté en réanimation au CHU de Caen, démontrant que chez des patients bronchitiques chroniques obstructifs sous ventilation mécanique nasale (VN) pour décompensation aiguë, que la VN améliorait les paramètres de déglutition de ces patients, mais que les manœuvres de déglutition pouvaient déclencher l'insufflation de ventilateur [5]. Ce désagrément n'était pas associé à des fausses routes détectables ou à d'autres complications [5]. Pour cette raison, nous avons mis au point un prototype, à partir d'un ventilateur existant (Elisée 150, ResMed), susceptible de ne plus se déclencher lorsque le patient commande un contacteur adaptable à son handicap, permettant ainsi à ce patient de déglutir sans appréhender une insufflation inadaptée du ventilateur au moment de la déglutition.

Objectif :

L'objectif principal de l'étude est de démontrer que la déglutition est plus aisée sous ventilation nasale (VN) adaptée qu'en ventilation spontanée (VS) sur une population neuromusculaire sous ventilation non invasive nocturne et diurne.

Méthodes :
Etude en cross-over, randomisée, en ouvert, les patients étant leur propre témoin. Deux conditions seront comparées : VS vs VN.
Pour chaque condition cinq enchaînements de 2 volumes différents d'eau (5 et 10 ml) minérale qui se succèderont suivis de 5 cuillères à café (5ml) de Danette (Danone)
L'évaluation consistera à mesurer la durée de la déglutition, le nombre de gorgées, le nombre de cycles respiratoires nécessaires et le pourcentage de gorgées suivies par une expiration. La sensation de dyspnée sera également évaluée.
Critères de sélection :
Patients adultes, atteints de pathologies neuromusculaires, ventilés au long cours la nuit et le jour de manière non invasive, à l'état stable au moment de l'étude.
Nombre de patients, centre :
Dix sujets seront inclus au cours de l'étude. Les sujets seront recrutés dans l'unité de ventilation à domicile du service de réanimation de l'hôpital Raymond Poincaré au cours de leur hospitalisation dans le cadre du suivi habituel de leur pathologie respiratoire.
Durée de l'étude : 24 mois

2 JUSTIFICATION SCIENTIFIQUE ET DESCRIPTION GÉNÉRALE DE LA RECHERCHE.

2.1 Description générale du dispositif

2.1.1 Ventilateur

Elisee 150 (ResMed) modifié (voir ci-dessous).

2.1.2 Contacteur

Nous avons récemment fait une étude de recherche clinique utilisant un ventilateur dont un des paramètres de réglage pouvaient automatiquement être modifiés à l'aide d'un contacteur sous le contrôle de patients neuromusculaires. L'objectif était d'optimiser la parole des patients par un réglage du ventilateur adapté à la phonation (mais pas à la situation de repos) et donc dès que le patient décide de parler il peut actionner le contacteur. Selon le même principe de contrôle du ventilateur par le patient, nous souhaitons que dès que le patient décide de déglutir, en actionnant le même contacteur, il puisse empêcher le ventilateur de générer une insufflation au moment de la déglutition. Cette marge de manœuvre donnée au patient est une sécurité supplémentaire. Il peut décider à tout instant d'interrompre la ventilation mécanique sans se débrancher du ventilateur, alors qu'antérieurement il était obligé d'adapter sa déglutition au rythme du ventilateur ce qui limitait l'utilisation du ventilateur au cours de l'alimentation, et nécessitait, en général, une alimentation en condition de ventilation spontanée.

2.2 Résumé des résultats des essais non cliniques et cliniques disponibles et pertinents

Voir le chapitre « contexte ». En dehors des travaux que nous avons publiés nous avons l'expérience clinique de patients qui mangent alors qu'ils sont sous ventilation nasale et qui se débrouillent pour déglutir au moment du temps expiratoire réglé sur le ventilateur. L'équipe du centre de rééducation d'Inkendaal, (Inkendaalstraat 1, B-1602 Vlezenbeek, Brussels, Belgium) nous a fait part de patients qui faisaient une hyperinflation pulmonaire mécanique par embout buccal avant de prendre un bol alimentaire par la bouche et d'avaler. Nous pensons qu'il est plus simple pour le patient de prendre par la bouche le bol alimentaire alors qu'il est encore sous ventilation mécanique nasale, et de mastiquer si nécessaire, puis de permettre au patient d'interrompre la ventilation mécanique au moment où il décide de déglutir.

2.3 Résumé des bénéfices et des risques prévisibles et connus

Nous nous attendons à une déglutition plus rapide, moins fragmentée, mieux synchronisée à la ventilation et moins dyspnéisante sous VN que sous VS.

Le dispositif utilisé étant sous le contrôle du patient au moyen d'un contacteur, il n'est attendu aucun effet secondaire. En effet, le sujet peut à tout moment reprendre la ventilation mécanique en arrêtant d'appuyer sur le contacteur.

2.4 Description de la population à étudier.

Les patients étudiés seront des patients adultes suivis pour une insuffisance respiratoire chronique d'origine neuromusculaire nécessitant une ventilation mécanique par voie non-invasive. Ils seront habituellement ventilés la nuit et le jour par voie nasale ou buccale avec une durée minimum de 14h/jour.

Les patients au moment de l'étude seront à l'état stable, à distance de tout épisode aigu de décompensation respiratoire.

3 OBJECTIFS DE LA RECHERCHE.

3.1 Objectif principal

Démontrer qu'une ventilation nasale adaptée améliore les paramètres de déglutition (durée, nombre de gorgées, nombre de cycles respiratoires nécessaires) des patients neuromusculaires insuffisants respiratoires.

3.2 Objectif(s) secondaire(s)

Démontrer qu'une ventilation nasale adaptée améliore la synchronisation ventilation-déglutition et la dyspnée induite par la déglutition des patients neuromusculaires insuffisants respiratoires.

4 CONCEPTION DE LA RECHERCHE.

4.1 Critère de jugement principal

L'efficacité de la déglutition évaluée par trois critères:

- Durée de la déglutition
- Nombre de gorgées par déglutition
- Nombre de cycles respiratoires nécessaires par déglutition

4.2 Critère(s) de jugement secondaire(s)

- Pourcentages de gorgées suivies par une expiration
- La sensation de dyspnée évaluée par l'Echelle de Borg

4.3 Plan expérimental - déroulement pratique

Il s'agit d'une étude en cross-over, randomisée, en ouvert, les patients étant leur propre témoin. Les patients seront recrutés au cours de leur bilan respiratoire systématique dans le pôle de ventilation à domicile du service réanimation de l'hôpital Raymond Poincaré. Ces bilans comportent des hospitalisations de 2 à 3 jours permettant de réaliser les examens évaluant l'état respiratoire et la ventilation de ces patients.

Les patients seront contactés le jour de leur arrivée, le protocole leur sera proposé et expliqué par un médecin investigateur, le formulaire de consentement leur sera remis.

Si les patients acceptent de participer à l'étude, celle-ci sera réalisée le lendemain avant la sortie programmée du patient. La durée d'hospitalisation ne sera donc pas prolongée pour la réalisation du protocole.

Le protocole ne comportant pas de changement de traitement ni de prise en charge pour le patient, il n'est pas prévu de visite de contrôle.

Déroulement de l’essai

L’évaluation propre à l’étude durera environ 1 heure 30. Celle-ci sera réalisée, au sein du service de réanimation médicale en présence des réanimateurs, avec à disposition un chariot d’urgence en cas d’évènement intercurrent.

Un enregistrement continu de la saturation transcutanée en oxygène et du pouls sera effectué au cours de la période d’évaluation clinique par un oxymètre (Ohmeda Biox, BOC Healthcare, Boulder, CO). Les patients seront explorés en position confortable assise, similaire à la position habituellement utilisée au cours des repas. Les tests seront débutés après une période de repos de dix minutes nécessaire à l’installation des différents capteurs. La tête est positionnée de manière neutre afin de ne pas influencer les paramètres de la déglutition [6] et d’assurer l’homogénéité des enregistrements. La position de la tête est repérée, par une mesure de la distance menton/sternum.

Le ventilateur utilisé au cours de l’évaluation sera réglé selon les paramètres habituellement utilisés par le patient et l’adéquation de la ventilation sera vérifiée avant de débuter l’évaluation de la déglutition. Le patient sera également familiarisé avec l’utilisation du contacteur. L’exploration de la déglutition comprendra cinq enchaînements de 2 volumes différents d’eau (5 et 10 ml) minérale qui se succèderont de manière randomisée. Les quantités d’eau minérale seront déposées dans l’oropharynx à l’aide d’une seringue de 20 ml. Chacun des patients bénéficiera d’un enregistrement en ventilation spontanée sans support ventilatoire, et des mêmes enregistrements sous ventilation mécanique non invasive par masque nasal. Chacune des situations se succéderont dans un ordre randomisé. Les sujets ne sont pas informés du volume à déglutir. Les consignes données au préalable sont, qu’une fois l’ordre donné, le sujet doit avaler comme à son habitude en essayant d’être le plus efficace possible.

Après une période de repos et en l’absence de survenue de fausses routes avec l’eau minérale, il sera effectué, chez les patients un enregistrement, en continu, de la déglutition de l’enchaînement de 5 cuillères à café de Danette (Danone®). Afin de ne pas être limité par des déficits d’ordre moteur, celui-ci sera administré par une tierce personne. Cet enregistrement sera réalisé sous ventilation et en l’absence de support ventilatoire. Les situations ventilatoires se succèderont dans un ordre randomisé.

Une évaluation de la dyspnée, chez les patients, sera effectuée après chaque condition de ventilation, à l’aide d’une échelle de Borg.

Etude de la déglutition :

Il s’agit d’une évaluation non invasive, validée dans la littérature [7], utilisée initialement pour mettre en évidence des troubles de la déglutition dans des pathologies musculaires et neurologiques [7-10].

Cette évaluation consiste en l’enregistrement simultané des paramètres suivants :

- L’activité électromyographique de surface des muscles sus hyoïdiens par des électrodes bipolaires adhésives collées sous le menton en regard du plancher buccal. Le signal est amplifié et filtré (bande passante de 45 à 55 Hertz), redressé et intégré.
- Le mouvement du larynx par un capteur piézoélectrique scotché à l’aide d’un pansement adhésif transparent type Tegaderm (3M Health Care, St Paul, USA) sur la ligne médiane entre le cartilage cricoïde et le cartilage thyroïde (en moyenne 2 cm au-dessus de la canule de trachéotomie pour les patients trachéotomisés). Le signal est amplifié et filtré (bande passante de 45 à 55 Hertz).

La corrélation des signaux EMG et laryngé permettra de distinguer pendant l’analyse les artéfacts des vraies déglutitions.

L’exploration de la respiration pendant la déglutition se fera par le recueil des paramètres suivants:

- Les mouvements respiratoires thoraciques et abdominaux sont enregistrés par pléthysmographie d’inductance (Respitrace ; Ambulatory Monitoring, Ardsley, NY). Les bandes thoraciques sont placées autour du thorax au-dessus de la ligne mamelonnaire, les bandes abdominales au niveau ombilical.
- Pendant les enregistrements réalisés sous ventilation non invasive la pression dans le circuit inspiratoire du ventilateur sera enregistré à l’aide d’un capteur différentiel de pression (Valydine MP 45 ± 100 cm H_2O, Californie, USA). De même, le débit délivré par le ventilateur sera mesuré par un pneumotachographe (Fleisch#1, Lausanne, Suisse) connecté à un capteur différentiel de pression (Valydine MP 45 ± 5 cm+H_2O, Californie, USA).

Système d’acquisition :

L’ensemble des signaux respiratoires et de déglutition sera enregistré simultanément par un système d’acquisition capable de digitaliser les signaux analogiques (MP150, Biopac System, Santa Barbara, CA) puis traité par le programme Acknowledge (Biopac System). Les données seront enregistrées sur un ordinateur, via un système analogue-digital (MP150, Biopac System, Santa Barbara, CA).

4.4 *Description des mesures prises pour réduire et éviter les biais*

Afin d’éviter les biais d’apprentissage au cours de la procédure, l’ordre dans lequel les deux conditions (VS et VN) seront présentées sera randomisé.

4.5 Durée de la recherche

Pour chaque patient, l’évaluation de chaque condition ne devrait pas excéder 30 minutes. Par conséquent l’ensemble de l’essai ne devrait pas excéder 1 heure 30 minutes.

La durée prévue de l’étude, comprenant le recrutement et l’inclusion des patients ainsi que l’analyse des données, est de 24 mois.

4.6 Description des règles d’arrêt définitif ou temporaire

Le dispositif utilisé étant sous contrôle du patient au moyen d’un contacteur, et cette étude étant dénuée de tout geste invasif, il ne devrait pas y avoir d’évènement indésirable grave. Dans le cas contraire la conduite à tenir sera de remettre le patient sous son ventilateur habituel et d’en référer au réanimateur de garde.

4.7 Identification de toutes les données à recueillir directement dans les cahiers d’observation, qui seront considérées comme des données source.

Renseignements généraux :

Date d’inclusion
Nom, prénom (premières lettres)
Sexe
Date de naissance, âge
Diagnostic de la pathologie neuromusculaire
Date d’hospitalisation
Durée moyenne d’un repas (évaluée par le patient et sa famille / évaluée au cours de l’hospitalisation)
Echelle de Norris bulbaire
Score de Mallampati

Traitements en cours :

Type de ventilateur/ modèle/version
Durée de ventilation/jour
Date de la VNI diurne
Modalité de ventilation :
  Pression inspiratoire
  Fréquence respiratoire
  Volume minute ou volume courant
  FiO2
  Trigger
  I:E

Examen clinique
Poids, taille ou envergure, index de masse corporelle.
Fréquence cardiaque
Saturation en oxygène
Fréquence respiratoire

Epreuves fonctionnelles respiratoires
VEMS
CVF
CVL
VEMS/CVF
VEMS/CVL
CRF
VR
CPT
Pi max
Pe max
SNIP

Gazométrie artérielle
FiO_2 ou débitd'O_2
PO_2
PCO_2
pH

Pour chaque situation (VS vs VN)
Fréquence cardiaque
Saturation en oxygène
Fréquence respiratoire

Confort respiratoire (Borg)

Durée de la déglutition
Nombre de gorgées par déglutition
Nombre de cycles respiratoires par déglutition
% de gorgées suivies par une expiration

5 SÉLECTION DES PERSONNES DE LA RECHERCHE

Les patients seront recrutés parmi les patients neuromusculaires ventilés au long cours au cours du sommeil et au cours de la veille, suivis pour leur prise en charge respiratoire par l'unité de ventilation à domicile du service de réanimation de l'hôpital Raymond Poincaré.

5.1 Critères d'inclusion

- Patients présentant une insuffisance respiratoire restrictive d'origine neurologique ou musculaire quelle qu'en soit l'étiologie, sans atteinte suprabulbaire associée.
- Homme ou femme d'âge ≥ à 18 ans.
- Hospitalisation dans le secteur de « Ventilation à Domicile » de l'Hôpital R. Poincaré.
- Bénéficiant d'un support ventilatoire par VNI nocturne et diurne avec au total plus de 14h/jour de VNI.
- Persistance d'une autonomie respiratoire d'au moins 1 heure dans la journée (permettant la prise des repas).
- Mode de ventilation : assistée contrôlée (modes en pression ou en volume)
- Patients à l'état stable au moment de l'étude
- Réalisation d'un examen médical préalable
- Patient ayant signé un consentement éclairé et écrit
- Test de grossesse négatif pour les femmes en âge de procréer ou ayant une contraception efficace

5.2 Critères de non-inclusion des personnes qui se prêtent à la recherche

- Refus du patient de participer à l'étude
- Incapacité à coopérer
- Instabilité hémodynamique
- Patient en cours de décompensation respiratoire
- Non affiliation à un régime de sécurité sociale (bénéficiaire ou ayant droit)
- Malade sous curatelle ou tutelle

5.3 Participation simultanée à une autre recherche, période d'exclusion

Il n'est pas prévu de période d'exclusion pour participer à une autre recherche biomédicale**.**

6 ARRÊT PRÉMATURÉ DE L'UTILISATION DU (DES) DISPOSITIF(S)

En cas de demande du patient ou de complication non prévue.

7 TRAITEMENT UTILISÉ AUTRE QUE LE DISPOSITIF FAISANT L'OBJET DE LA RECHERCHE

Aucune modification des traitements de fond ni de la prise en charge n'est prévue pendant le protocole. Aucun autre traitement associé au protocole n'est prévu. Par conséquent aucun suivi spécifique n'est prévu au terme du protocole.

8 EVALUATION DE LA PERFORMANCE

Les paramètres d'évaluation de la performance seront :

Efficacité sur la déglutition

- Durée de la déglutition
- Nombre de gorgées par déglutition
- Nombre de cycles respiratoires par déglutition
- % de gorgées suivies par une expiration

➢ Confort de la déglutition (EVA)

Tolérance
Les paramètres respiratoires suivants seront recueillis avant et au terme de chaque lecture dans les différentes conditions

➢ Fréquence cardiaque
➢ Saturation en oxygène
➢ Fréquence respiratoire
➢ Confort respiratoire (Borg)

Utilisation du contacteur

➢ Dans chaque situation (durée d'utilisation du contacteur/durée de la déglutition)
➢ Dans chaque situation : (nombre de commutations du contacteur)/(unité de temps)
➢ Utilité du contacteur (EVA)
➢ Intérêt de la technique (oui/non/sans opinion)

9 EVALUATION DE LA SÉCURITÉ

9.1 Description des paramètres d'évaluation de la sécurité

Les paramètres habituels de ventilation des patients n'étant pas modifiés, il n'est pas attendu d'événement indésirable grave

En cas de mauvaise tolérance de la VN, celle-ci serait remplacée par conditions habituelles de ventilation mécanique avec le ventilateur habituel du patient.

L'ensemble de l'évaluation sera réalisé en présence d'un médecin familier avec les techniques de ventilation.

La tolérance respiratoire de l'évaluation sera surveillée par un monitoring continu de la saturation percutanée en oxygène, la fréquence cardiaque et la fréquence respiratoire.

- **Evènement indésirable**

Toute manifestation nocive survenant chez une personne qui se prête à une recherche biomédicale que cette manifestation soit liée ou non à tout élément expérimental de la recherche et ce qu'il s'agisse des actes pratiqués ou des produits utilisés.

- **Effet indésirable**

Toute réaction nocive et non désirée à tout élément expérimental de la recherche et ce qu'il s'agisse des actes pratiqués ou des produits utilisés.

- **Evènement ou effet indésirable grave**

Tout évènement ou effet indésirable qui entraîne la mort, met en danger la vie de la personne qui se prête à la recherche, nécessite une hospitalisation ou la prolongation de l'hospitalisation, provoque une incapacité ou un handicap importants ou durables, ou bien se traduit par une anomalie ou une malformation congénitale.

- **Effet indésirable inattendu**

Tout effet indésirable dont la nature, la sévérité ou l'évolution ne concorde pas avec les

informations figurant dans les référentiels reconnus par les autorités.
 Fait nouveau

Toute nouvelle donnée de sécurité, pouvant conduire à une réévaluation du rapport des bénéfices et des risques de la recherche ou qui pourrait être suffisant pour envisager des modifications des documents relatifs à la recherche, de la conduite de la recherche ainsi que, le cas échéant, dans l'utilisation du produit.

9.2 Méthodes et calendrier pour mesurer, recueillir et analyser les paramètres d'évaluation de la sécurité

Comité de pilotage

Il sera constitué des initiateurs cliniciens du projet, du biostatisticien en charge du projet, des représentants du promoteur et du CIC-IT nommés pour cette recherche.
Il définira l'organisation générale et le déroulement de la recherche et coordonnera les informations. Il déterminera initialement la méthodologie et décidera en cours de recherche des conduites à tenir dans les cas imprévus, surveillera le déroulement de la recherche en particulier sur le plan de la tolérance et des évènements indésirables.

9.3 Procédures mises en place en vue de l'enregistrement et de la notification des événements indésirables

9.3.1 Evènements indésirables non graves :

Tout événement indésirable - non grave suivant la définition précédente - observé lors de la recherche et dans ses suites devra être reporté dans le cahier d'observation dans la section prévue à cet effet.
Un seul évènement doit être reporté par item. L'évènement peut correspondre à un symptôme, un diagnostic ou à un résultat d'examen complémentaire jugé significatif. Tous les éléments cliniques ou para-cliniques permettant de décrire au mieux l'évènement correspondant doivent être reportés.

9.3.2 Evènements indésirables graves (EIG) :

Le formulaire de déclaration d'un événement indésirable grave, validé pour la recherche, est inclus dans le protocole en annexe.
L'investigateur est tenu de notifier **immédiatement** au promoteur tous les événements indésirables graves.
L'investigateur complète le formulaire de déclaration d'évènement indésirable grave (du cahier d'observation de la recherche) et l'envoie au promoteur par fax au 01 46 97 16 94 et ce, dans les 48 heures
L'investigateur doit également informer le CIC-IT en charge de la recherche de la survenue de l'EIG.
Pour chaque évènement indésirable grave, **l'investigateur devra émettre un avis sur le lien de causalité de l'évènement avec tout élément expérimental de la recherche et ce qu'il s'agisse des actes pratiqués ou des produits utilisés.**
L'obtention d'informations relatives à la description et l'évaluation d'un évènement indésirable peuvent ne pas être possibles dans le temps imparti pour la déclaration initiale.

Aussi, l'évolution clinique ainsi que les résultats des éventuels bilans cliniques et des examens diagnostiques et/ou de laboratoire, ou toute autre information permettant une analyse adéquate du lien de causalité seront rapportés :

- soit sur la déclaration initiale d'EIG s'ils sont immédiatement disponibles,

- soit ultérieurement et le plus rapidement possible, en envoyant par fax une nouvelle déclaration d'EIG complétée (et en précisant qu'il s'agit d'un suivi d'EIG déclaré et le numéro de suivi).

Toutes les déclarations faites par les investigateurs devront identifier chaque sujet participant à la recherche **par un numéro de code unique** attribué à chacun d'entre eux.

En cas de décès notifié d'un sujet participant à la recherche**, l'investigateur communiquera au promoteur tous les renseignements complémentaires demandés** (compte-rendu d'hospitalisation, résultats d'autopsie…).

Tout fait nouveau survenu dans la recherche ou dans le contexte de la recherche, provenant de données de la littérature ou de recherches en cours, devra être notifié au promoteur.

- Déclaration des évènements indésirables graves aux Autorités de Santé

Elle sera assurée par le promoteur, après évaluation de la gravité de l'évènement indésirable, du lien de causalité avec l'élément expérimental de la recherche et ce qu'il s'agisse des actes pratiqués ou des produits utilisés, ainsi que du caractère inattendu des effets indésirables.

Toutes les suspicions d'effet indésirable grave inattendu seront déclarées par le promoteur aux autorités compétentes dans les délais légaux.

Toute donnée de sécurité ou tout fait nouveau qui pourrait modifier significativement l'évaluation du rapport des bénéfices et des risques de l'élément expérimental de la recherche (et ce qu'il s'agisse des actes pratiqués ou des produits utilisés) ou de la recherche, ou qui pourrait conduire à envisager des modifications concernant la conduite de la recherche, sera transmise par le promoteur aux autorités compétentes, au Comité de Protection des Personnes et aux investigateurs de la recherche.

Par exemple :

a) toute augmentation cliniquement significative de la fréquence d'apparition d'un effet indésirable grave attendu ;

b) des suspicions d'effet indésirable grave inattendu survenus chez des participants ayant terminé l'essai et qui sont notifiés par l'investigateur au promoteur, ainsi que des rapports de suivi éventuels ;

c) tout fait nouveau concernant le déroulement de l'essai clinique, lorsque ce fait nouveau est susceptible de porter atteinte à la sécurité des participants. A titre d'exemple :

- un événement indésirable grave susceptible d'être lié aux investigations et aux procédures de diagnostic de l'essai et qui pourrait modifier le déroulement de cet essai,
- un risque significatif pour la population de l'essai comme par exemple un manque d'efficacité de l'élément expérimental utilisé dans le traitement d'une maladie mettant en jeu le pronostic vital,
- des résultats significatifs de sécurité issus d'une étude menée chez l'animal récemment terminée (telle qu'une étude de carcinogénicité),
- un arrêt anticipé ou une interruption temporaire pour des raisons de sécurité d'un essai conduit avec le même élément expérimental dans un autre pays,

d) les recommandations du comité de surveillance indépendant [Data Monitoring Committee

(DMC) ou Data Safety Monitoring Board (DSMB)], le cas échéant, si elles sont pertinentes pour la sécurité des personnes,
e) tout effet indésirable grave inattendu transmis au promoteur par un autre promoteur d'un essai clinique mené dans un pays tiers portant sur le même élément expérimental.

9.4. Modalités et durée du suivi des personnes suite à la survenue d'évènements indésirables

Tout patient présentant un évènement indésirable doit être suivi jusqu'à la résolution ou la stabilisation de celui-ci.

- Si l'évènement n'est pas grave, l'évolution en sera notée sur la page correspondante du cahier d'observation à la section prévue à cet effet.
- Si l'évènement est grave, un suivi d'EIG sera envoyé au promoteur.

10 STATISTIQUES

10.1 Nombre prévu de personnes à inclure dans la recherche, et nombre prévu de personnes dans chaque lieu de recherches avec sa justification statistique.

Dix sujets seront inclus au cours de l'étude. Une étude antérieure du service dans le même domaine de recherche effectuée sur 7 patients et comparant 2 conditions de ventilation avait mis en évidence des différences hautement significatives [11]. Par conséquent 10 patients devraient suffire pour atteindre des différences significatives.

Les sujets seront recrutés dans l'unité de ventilation à domicile du service de réanimation de l'hôpital Raymond Poincaré au cours de leur hospitalisation dans le cadre du suivi habituel de leur pathologie respiratoire.

10.2 Description des méthodes statistiques prévues, y compris du calendrier des analyses intermédiaires prévues.

Il s'agit d'une étude d'efficacité et l'analyse principale sera faite en intention de traiter sur tous les sujets randomisés selon la stratégie prescrite à la randomisation, indépendamment du traitement effectivement reçu et des écarts au protocole. Une cause d'exclusion retenue est le retrait du consentement du patient.

Les données seront résumées par groupe, par les statistiques descriptives usuelles suivant la nature de la variable :

- Variables quantitatives : n, minimum, maximum, médiane, moyenne, écart-type, étendue et étendue interquartile,
- Variable qualitatives : n, effectifs, pourcentages.

Les méthodes statistiques utilisées seront des comparaisons de pourcentages, de moyennes et/ou de médianes des différences par rapport à zéro.
Les critères quantitatifs (résultats obtenus avec les différents dispositifs d'amélioration de la parole) seront comparés par une analyse de variance pour mesures répétées à deux facteurs conditions (VN, VS), matériel dégluti (5 ml d'eau, 10 ml d'eau, 5 cc de Danette).

L'analyse de la tolérance sera effectuée en intention de traiter.
Les événements indésirables seront décrits par leur fréquence et présentés en fonction du bras de traitement, du type d'événement, de la relation de causalité (certaine, probable, possible, non lié)

et de leur intensité.

Les tests seront effectués avec un risque de première espèce □ de 5 % en formulation bilatérale.

Les analyses statistiques seront réalisées à l’aide des logiciels R et S-plus au sein du CIC-IT.

10.3 Méthodes de prise en compte des données manquantes, inutilisées ou non valides

On vérifiera que le taux de données manquantes et/ou inutilisables est le même en fonction du groupe de traitement. S’il s’avère néanmoins qu’il y a une différence, la causalité en sera recherchée.
La prise en compte des données manquantes sur le critère principal sera réalisée en fonction de la complexité des situations (remplacement par la dernière valeur disponible, remplacement par l’effet moyen du groupe…..). Une analyse de sensibilité sera réalisée.

10.4 Analyse intermédiaire

Il n’y a pas d’analyse intermédiaire programmée

10.5 Choix des personnes à inclure dans l’analyse.

Le statut des patients : sélectionnés, randomisés, évalués en intention de traiter (ITT), perdus de vue ou sortis d’études, déviations au protocole, sera décrit et comparé par bras.

11 DROIT D’ACCÈS AUX DONNÉES ET DOCUMENTS SOURCE

Les personnes ayant un accès direct conformément aux dispositions législatives et réglementaires en vigueur, notamment les articles L.1121-3 et R.5121-13 du code de la santé publique (par exemple, les investigateurs, les personnes chargées du contrôle de qualité, les moniteurs, les assistants de recherche clinique, les auditeurs et toutes personnes appelées à collaborer aux essais) prennent toutes les précautions nécessaires en vue d'assurer la confidentialité des informations relatives aux médicaments expérimentaux, aux essais, aux personnes qui s'y prêtent et notamment en ce qui concerne leur identité ainsi qu’aux résultats obtenus. Les données collectées par ces personnes au cours des contrôles de qualité ou des audits sont alors rendues anonymes.

12 CONTRÔLE ET ASSURANCE DE LA QUALITÉ

La recherche sera encadrée selon les procédures opératoires standard du promoteur. Le déroulement de la recherche dans les centres investigateurs et la prise en charge des sujets sera faîte conformément à la déclaration d’Helsinki et les Bonnes Pratiques en vigueur.

12.1 Procédures de monitoring

Les ARC représentants du promoteur effectueront des visites des centres investigateurs au rythme correspondant au schéma de suivi des patients dans le protocole, aux inclusions dans les différents centres et au niveau de risque qui a été attribué à la recherche.

- Visite d‘ouverture de chaque centre : avant inclusion, pour une mise en place du protocole et prise de connaissance avec les différents intervenants de la recherche biomédicale.

- Lors des visites suivantes, les cahiers d'observation seront revus au fur et à mesure de l'état d'avancement de la recherche par les ARC. L'investigateur principal de chaque centre ainsi que les autres investigateurs qui incluent ou assurent le suivi des personnes participant à la recherche

s’engagent à recevoir les ARC à intervalles réguliers.
Lors de ces visites sur site et en accord avec les Bonnes Pratiques Cliniques, les éléments suivants seront revus :
 Respect du protocole et des procédures définies pour la recherche,
 Vérification des consentements éclairés des patients
 Examen des documents source et confrontation avec les données reportées dans le cahier d’observation quant à l’exactitude, les données manquantes.

- Visite de fermeture : récupération des cahiers d’observation, bilan à la pharmacie, documents de la recherche biomédicale, archivage.

12.2 Transcription des données dans le cahier d’observation

Toutes les informations requises par le protocole doivent être fournies dans le cahier d’observation et une explication donnée par l’investigateur pour chaque donnée manquante.
Les données devront être transférées dans les cahiers d'observation au fur et à mesure qu'elles sont obtenues qu'il s'agisse de données cliniques ou para cliniques. Les données devront être copiées de façon nette et lisible à l'encre noire dans ces cahiers (ceci afin de faciliter la duplication et la saisie informatique).

Les données erronées, dépistées sur les cahiers d'observation seront clairement barrées et les nouvelles données seront copiées sur le cahier avec les initiales et la date par le membre de l'équipe de l'investigateur qui aura fait la correction.
L'anonymat des sujets sera assuré par la mention de la première lettre du nom, la première lettre du prénom et le numéro d’inclusion du sujet sur tous les documents nécessaires à la recherche.
Les données informatisées sur un fichier seront déclarées à la CNIL selon la procédure adaptée au cas.

13 CONSIDÉRATIONS ÉTHIQUES

Le promoteur est défini par la loi 2004-806 du 9 août 2004. Dans cette recherche, **ADEP ASSISTANCE est le promoteur**.

Avant de démarrer la recherche, chaque investigateur fournira au représentant du promoteur de la recherche une copie de son curriculum vitæ personnel daté et signé, comportant son numéro d’inscription à l’Ordre des Médecins et son numéro ADELI

13.1 Demande d’autorisation auprès de l’autorité compétente

Pour pouvoir démarrer la recherche, le promoteur doit soumettre un dossier de demande d’autorisation auprès de l'autorité compétente l’Afssaps. L'autorité compétente, définie à l'article L. 1123-12, se prononce au regard de la sécurité des personnes qui se prêtent à une recherche biomédicale, en considérant notamment la sécurité et la qualité des produits utilisés au cours de la recherche conformément, le cas échéant, aux référentiels en vigueur, leur condition d'utilisation et la sécurité des personnes au regard des actes pratiqués et des méthodes utilisées ainsi que les modalités prévues pour le suivi des personnes.

13.2 Demande d’avis au Comité de Protection des Personnes

En accord avec l'article L.1123-6 du Code de Santé Publique, le protocole de recherche doit être soumis par le promoteur à un Comité de Protection des Personnes. L'avis de ce comité est notifié à l’autorité compétente par le promoteur avant le démarrage de la recherche.

Le promoteur doit être informé de tout projet de modification du protocole par l’investigateur coordonnateur.
Les modifications devront être qualifiées en substantielles ou non.

Une modification substantielle est une modification susceptible, d'une manière ou d'une autre, de modifier les garanties apportées aux personnes qui se prêtent à la recherche biomédicale (modification d'un critère d'inclusion, prolongation d'une durée d'inclusion, participation de nouveaux centres,…).

Après le commencement de la recherche, toute modification substantielle de celle-ci à l'initiative du promoteur doit obtenir, préalablement à sa mise en oeuvre, un avis favorable du comité et une autorisation de l'autorité compétente. Dans ce cas, si cela est nécessaire, le comité s'assure qu'un nouveau consentement des personnes participant à la recherche est bien recueilli.

Par ailleurs, toute extension de la recherche (modification profonde du schéma thérapeutique ou des populations incluses, prolongation des traitements et ou des actes thérapeutiques non prévus initialement dans le protocole) devra être considérée comme une nouvelle recherche.

Toute modification substantielle devra faire l'objet par le promoteur après paiement d'une taxe d'une demande d'autorisation auprès de l'Afssaps et/ou d'une demande d'avis du CPP.

13.3 Déclaration CNIL

La loi prévoit que la déclaration du fichier informatisé des données personnelles collectées pour la recherche doit être faite avant le début effectif de la recherche.

Une méthodologie de référence spécifique au traitement de données personnelles opéré dans le cadre des recherches biomédicales définies par la loi 2004-806 du 9 août 2004 car entrant dans le champ des articles L.1121-1 et suivants du Code de Santé Publique a été établie par la CNIL en janvier 2006.

Cette méthodologie permet une procédure de déclaration simplifiée lorsque la nature des données recueillies dans la recherche est compatible avec la liste prévue par la CNIL dans son document de référence.

Lorsque le protocole bénéficie d'un contrôle qualité des données par un ARC représentant le promoteur et qu'il entre dans le champ d'application de la procédure simplifiée CNIL, l'ADEP Assistance en qualité de promoteur demandera au responsable du fichier informatique de s'engager par écrit sur le respect de la méthodologie de référence MR06001 simplifiée.

13.4 Note d'information et Consentement éclairé

Aucune recherche biomédicale ne peut être pratiquée sur une personne sans son consentement libre et éclairé, recueilli après que lui a été délivrée l'information prévue à l'article L. 1122-1.

Le consentement est donné par écrit ou, en cas d'impossibilité, attesté par un tiers. Ce dernier doit être totalement indépendant de l'investigateur et du promoteur.

13.5 Rapport final de la recherche

Le rapport final de la recherche sera écrit en collaboration par le coordonnateur et le biostatisticien pour cette recherche. Ce rapport sera soumis à chacun des investigateurs pour avis. Une fois qu'un consensus aura été obtenu, la version finale devra être avalisée par la signature de chacun des investigateurs et adressée au promoteur dans les meilleurs délais après la fin effective de la recherche. Un rapport rédigé selon le plan de référence de l'autorité compétente doit être transmis à l'autorité compétente ainsi qu'au CPP dans un délai de un an, après la fin de la recherche, s'entendant comme la dernière visite de suivi du dernier sujet inclus. Ce délai est rapporté à 90 jours en cas d'arrêt prématuré de la recherche.

14 TRAITEMENT DES DONNÉES ET ARCHIVAGE

Les documents d'une recherche entrant dans le cadre de la loi sur les recherches biomédicales doivent être archivés par toutes les parties pendant une durée de 15 ans après la fin de la recherche.

Cet archivage indexé comporte :
- Les copies de courrier d'autorisation de l'Afssaps et de l'avis obligatoire du CPP
- Les versions successives du protocole (identifiées par le n° de version et la date de version),
- Les courriers de correspondance avec le promoteur,
- Les consentements signés des sujets sous pli cacheté (dans le cas de sujets mineurs signés par les titulaires de l'autorité parentale) avec la liste ou registre d'inclusion en correspondance,
- Le cahier d'observation complété et validé de chaque sujet inclus,
- Toutes les annexes spécifiques à l'étude,
- Le rapport final de l'étude provenant de l'analyse statistique et du contrôle qualité de l'étude (double transmis au promoteur).
- Les certificats d'audit éventuels réalisés au cours de la recherche

La base de données ayant donné lieu à l'analyse statistique doit aussi faire l'objet d'archivage par le responsable de l'analyse (support papier ou informatique).

15 FINANCEMENT ET ASSURANCE

15.1 Assurance

ADEP Assistance est le promoteur de cette recherche. En accord avec la loi sur les recherches biomédicales, elle a pris une assurance auprès de la compagnie Biomedi Insure pour toute la durée de la recherche, garantissant sa propre responsabilité civile ainsi que celle de tout intervenant (médecin ou personnel impliqué dans la réalisation de la recherche) (loi n°2004-806, Art L.1121-10 du CSP).

ADEP Assistance se réserve le droit d'interrompre la recherche à tout moment pour des raisons médicales ou administratives; dans cette éventualité, une notification sera fournie à l'investigateur.

15.2 Engagement scientifique

Chaque investigateur s'engagera à respecter les obligations de la loi et à mener la recherche selon les B.P.C., en respectant les termes de la déclaration d'Helsinki en vigueur. Pour ce faire, un exemplaire de l'engagement scientifique daté et signé par chaque investigateur principal de chaque service clinique d'un centre participant sera remis au représentant du promoteur.

16 RÈGLES DE PUBLICATION

Le promoteur est propriétaire des données et aucune utilisation ou transmission à un tiers ne peut être effectuée sans son accord préalable.

Seront premiers signataires des publications, les personnes ayant réellement participé à l'élaboration du protocole et son déroulement ainsi qu'à la rédaction des résultats.

ADEP Assistance doit être mentionnée comme étant le promoteur de la recherche biomédicale et comme soutien financier le cas échéant.

17 BIBLIOGRAPHIE

1. Willig TN, Paulus J, Lacau Saint Guily J, Beon C, Navarro J: **Swallowing problems in neuromuscular disorders**. *Arch Phys Med Rehabil* 1994, **75**(11):1175-1181.

2. Terzi N, Orlikowski D, Aegerter P, Lejaille M, Ruquet M, Zalcman G, Fermanian C, Raphael JC, Lofaso F: **Breathing-swallowing interaction in neuromuscular patients: a physiological evaluation**. *Am J Respir Crit Care Med* 2007, **175**(3):269-276.

3. Terzi N, Prigent H, Lejaille M, Falaize L, Annane D, Orlikowski D, Lofaso F: **Impact of tracheostomy on swallowing performance in Duchenne muscular dystrophy**. *Neuromuscul Disord* 2010, **20**(8):493-498.

4. Bonanno PC: **Swallowing dysfunction after tracheostomy**. *Ann Surg* 1971, **174**(1):29-33.
5. Terzi N: **Evaluation des troubles de la déglutition et des interactions ventilation/déglutition en cas d'insuffisance respiratoire chronique respiratoire d'origine neuromusculaire**. *Thèse de Sciences de la Vie et de la Santé.* Versailles: Université de Versailles Saint-Quentin-en-Yvelines; 2010.
6. Ertekin C, Keskin A, Kiylioglu N, Kirazli Y, On AY, Tarlaci S, Aydogdu I: **The effect of head and neck positions on oropharyngeal swallowing: a clinical and electrophysiologic study**. *Arch Phys Med Rehabil* 2001, **82**(9):1255-1260.
7. Ertekin C, Yuceyar N, Aydogdu I: **Clinical and electrophysiological evaluation of dysphagia in myasthenia gravis**. *J Neurol Neurosurg Psychiatry* 1998, **65**(6):848-856.
8. Ertekin C, Aydogdu I, Yuceyar N, Kiylioglu N, Tarlaci S, Uludag B: **Pathophysiological mechanisms of oropharyngeal dysphagia in amyotrophic lateral sclerosis**. *Brain* 2000, **123 (Pt 1)**:125-140.
9. Ertekin C, Tarlaci S, Aydogdu I, Kiylioglu N, Yuceyar N, Turman AB, Secil Y, Esmeli F: **Electrophysiological evaluation of pharyngeal phase of swallowing in patients with Parkinson's disease**. *Mov Disord* 2002, **17**(5):942-949.
10. Ertekin C, Yuceyar N, Aydogdu, Karasoy H: **Electrophysiological evaluation of oropharyngeal swallowing in myotonic dystrophy**. *J Neurol Neurosurg Psychiatry* 2001, **70**(3):363-371.
11. Terzi N, Prigent H, Lejaille M, Falaize L, Annane D, Orlikowski D, Lofaso F: **Impact of tracheostomy on swallowing performance in Duchenne muscular dystrophy**. *Neuromuscul Disord*.
