# Supplementary material for: Noninvasive Mechanical Ventilation Improves Breathing-Swallowing Interaction of Ventilator Dependent Neuromuscular Patients: A Prospective Crossover Study
Source: PLoS One. 2016 Mar 3;11(3):e0148673. doi: 10.1371/journal.pone.0148673 (PMC4777441; doi:10.1371/journal.pone.0148673)
Supplement: S2 Protocol — (PDF) [file pone.0148673.s003.pdf]

OptiDEG

Evaluation of the efficiency of nasal non invasive ventilation on the optimisation of the swallowing of neuromuscular ventilated patients

Pilot cross-over, randomised, open study versus spontaneous breathing.

Version n° 3 - Nov 11th 2012

Cordinator Investigator:
Dr Hélène Prigent
Physiologie Explorations Fonctionnelles, Centre d'Investigation Clinique et d'Innovation Technologique, Hôpital Raymond Poincaré, Garches, France.
Tel : 01 47 10 79 11 Fax : 01 47 10 79 43
Email : helene.prigent@rpc.aphp.fr

Scientific comittee:
Principal : Pr Frédéric Lofaso
Physiologie Explorations Fonctionnelles, Centre d'Investigation Clinique et d'Innovation Technologique, Hôpital Raymond Poincaré, Garches, France.
Tel : 01 47 10 79 40 Fax : 01 47 10 79 43
Email : f.lofaso@rpc.aphp.fr
Associated : Dr. Nicolas Terzi
Service de réanimation médicale, CHU de Caen, Avenue de la Côte-de-Nacre, 14033 Caen Cedex, France
Tél : 02.31.06.52.68 Fax : 02.31.06.49.96
Email: terzi-n@chu-caen.fr

Investigators :
Dr David Orlikowski
Unité de Ventilation à domicile –Service de Réanimation médicale
Hôpital Raymond Poincaré, Garches
Tel : 01 47 10 77 76 Fax : 01 47 10 44 46
Email : david.orlikowski@rpc.aphp.fr

Associated team :
Speech therapist
Marine Garguilo
Centre d'Investigation Clinique et d'Innovation Technologique
Physiologie Explorations Fonctionnelles,
Hôpital Raymond Poincaré, Garches.
Tel : 01 47 10 46 15 Fax : 01 47 10 79 33
Email : marine.garguilo@rpc.aphp.fr

Study Promotor :
ADEP assistance

Mme Annie Leroy
2 rue Benoît Melon 92150 Suresnes
Tel : 01 46 97 12 87 Fax : 01 46 97 16 94
E-mail : adepassistance@adep.asso.fr

Methodology and management center :
Centre d'Investigation Clinique et d'Innovation Technologique (CIC-IT) :

Dr Frédéric Barbot
Hôpital Raymond Poincaré-104 avenue Raymond Poincaré-92380 Garches
Tél : 01.47.10.46.15 Fax : 01.47.10.46.33
Email : frederic.barbot@apr.aphp.fr

Page de SIGNATURE D'UN PROTOCOLE de recherche biomédicale
par l'investigateur COORDONNATEUR et le représentant du PROMOTEUR

Recherche Biomédicale **N° ANSM : 2011-A00771-40**

Titre *:* **«Evaluation de l'efficacité de la ventilation nasale sur l'optimisation de la déglutition chez les sujets neuromusculaires ventilés**

Etude pilote en cross-over, randomisée, en ouvert, versus ventilation spontanée»

Version N° 3 du : 12/11/2012

<u>L'investigateur coordonnateur :</u>

Dr Hélène Prigent
Service de :
Physiologie Explorations Fonctionnelles, Centre d'Investigation Clinique et d'Innovation Technologique, Hôpital Raymond Poincaré, Garches
Hôpital Universitaire Raymond Poincaré
104 bd Raymond Poincaré – 92380 Garches

L'investigateur coordonnateur autorise également la diffusion de son identité et de ses coordonnées dans le répertoire des recherches biomédicales

Date : 07/11/2012

Signature :

Responsables scientifiques :

Pr Frédéric Lofaso
Physiologie Explorations Fonctionnelles, Centre d'Investigation Clinique et d'Innovation Technologique, Hôpital Raymond Poincaré, Garches.

Date : 12/11/2012

Signature :

<u>Le promoteur</u> :

ADEP assistance
Mme Annie Leroy
2 rue Benoît Melon 92150 Suresnes
Tel : 01 46 97 12 87 Fax : 01 46 97 16 94

Date : 14/12/2012

ADE[illegible]ISTANCE S.A.
2 [illegible]
[illegible]SURESNES
Tél. 01 46 97 12 87
Fax 01 46 97 16 94
Au capital de 12 500 000 €
SIREN : 487 778 151 RCS Paris

Signature :

1 PROJECT SUMMARY. 6
2 SCIENTIFIC JUSTIFICATION AND GENERAL DESCRIPTION GÉNÉRALE OF RESEARCH 7
2.1 GENERAL DESCRIPTION OF DEVICE 7
2.2 SUMMARY OF PREVIOUS AVAILABLE AND RELEVANT NON-CLINICAL AND CLINICAL STUDIES……….. 7
2.3 SUMMARY OF EXPECTED BENEFITS AND RISKS.……………………… 7
2.4 DESCRIPTION OF STRUDY POPULATION.………….. 7
3 RESEARCH OBJECTIVES……….. 8
3.1 MAIN OBJE CTIVE 8
3.2 SECONDARY OBJECTIVE(S).. 8
4 RESEARCH CONCEPT……………….. 8
4.1 PRIMARY STUDY ENDPOINT 8
4.2 SECONDARY STUDY ENDPOINT(S)... 8
4.3 EXPERIMENTAL PLAN – PRACTICAL LAYOUT…….. 8
4.4 DESCRIPTION OF THE STEPS TAKEN TO REDUCE AND TO AVOID BIAS 9
4.5 STUDY DURATION 10
4.6 DESCRIPTION OF THE RULES FOR DEFINITIVE OR TEMPORARY STUDY INTERRUPTION................. 10
4.7 DESCRIPTION OF THE DATA DIRECTLY COLLECTED FROM THE OBSERVATION HANDBOOK AND WHICH WILL BE CONSIDERED AS SOURCE DATA. 10
5 SELECTION OF STUDY POPULATION 11
5.1 INCLUSION CRITERIA…….. 11
5.2 EXCLUSION CRITERIA………………………………………………………….......................... 11
5.3 RULES CONCERNING SIMULATNEOUS PARTICIPATION TO ANOTHER RESEARCH STUDY, EXCLUSION PERIOD.. 11
6 PREMATURE STOP OF DEVICE(S) USE……………………………………................... 12
7TREATMENTS USED OUTSIDE OF THE DEVICE CONCERNED BY THE STUDY………………………………………………………………………………12
8 PERFORMANCE EVALUATION ……….. 12
9 SECURITY EVALUATION ………... 12
9.1 DESCRIPTION OF PARAMETERS FOR SECURITY EVALUATION………….................................. 12
9.2 METHODS AND CALENDAR FOR MEASUREMENT, COLLECTION AND ANALYSIS OF PARAMETERS FOR SECURITY EVALUATION……………………………………………………….. 13
9.3 PROCEDURES ADOPTED FOR RECORDING AND REPORTING ADVERSE EVENTS.................... 13
9.4 PROCEDURES AND FOLLOWUP OF PARTICIPANTS FOLLOWING ADVERSE EVENTS ……….. 15
10 STATISTICS... 15
10.1 NUMBER OF PARTICIPANTS TO BE INCLUDED IN THE STUDY, AND NUMBER OF PATIENTS IN EACH STUDY CENTER WITH THE STATISTICAL JUSTIFICATION. 15
10.2 DESCRIPTION OF PLANNED STATISTICAL ANALYSIS, INCLUDING CALENDAR OF PLANNED INTERMEDIARY ANALYSIS.. 15
10.3 METHODS FOR MISSING ,UNUSED OR NON-VALID DATA MANAGMENT…............................. 16
10.4 INTERMEDIARY ANALYSIS 16

10.5 CHOICE OF PERSONS TO BE INCLUDED IN THE ANALYSIS.. 16
11 RIGHT OF ACCESS TO DATA AND SOURCE DOCUMENTS……............................. 16
12 QUALITY CONTROL AND GUARANTEES……….. 16
12.1 MONITORING PROCEDURES ……... 16
12.2 DATA TRANSCRIPTION IN THE OBSERVATION HANDBOOK…………………..................................... 17
13 ETHICAL CONSIDERATIONS.. 17
13.1 AUTHORIZATION REQUEST TO THE COMPETENT AUTHORITY 17
13.2 REQUEST FOR OPINION WITH THE STUDY PARTICIPANT PROTECTION COMMITTEE...................... 17
13.3 CNIL NOTIFICATION 18
13.4 INFORMATION NOTE AND INFORMED CONSENT.. 18
13.5 FINAL RESEARCH REPORT 18
14 DATA TREATMENT AND STORAGE….. 18
15 FUNDING AND INSURANCE 19
15.1 INSURANCE 19
15.2 SCIENTIFIC COMMITMENT.. 19
16 RULES OF PUBLICATION 20
17 BIBLIOGRAPHY 20

1 PROJECT SUMMARY.

Context :

Neuromuscular disorders may be associated to swallowing impairment due to weakness of the upper airways muscles involved in swallowing [1]. We showed that respiratory failure may participate in swallowing disorder and that, when tracheostomy is performed, as it allows to apply mechanical ventilation during swallowing, it improved swallowing performances [2-3] although by itself tracheostomy has been largely reported as deleterious to swallowing [4]. The aim now is to determine whether nasal non-invasive ventilation may be beneficial for swallowing, pending a few adjustments to allow a good synchronization between ventilation and swallowing. This could help to prevent tracheostomy or gastrostomy due to swallowing impairment and/or malnutrition. Improving swallowing during mechanical ventilation requires technically optimizing synchronization between the patient and the ventilator during swallowing. On this subject, a previous study was initiated in the intensive care unit of the Caen teaching hospital, demonstrating that chronic obstructive pulmonary disease (COPD) patients under nasal non-invasive ventilation (NNIV) improved their swallowing parameters while ventilated but the movements associated with swallowing were liable to trigger insufflation from the ventilator[5]. This side-effect was not associated with clinically detectable aspiration or any other complication [5]. Therefore, we developed a prototype from an already commercialized ventilator (Elisée 150, ResMed), liable to not be triggered when controlled by the patient through switch adapted to the patient's motor handicap, allowing the patient to swallow without risking receiving an inadequate insufflation from the ventilator while swallowing.

Objective:

The main objective of the study is to demonstrate that swallowing is easier under adapted nasal non-invasive ventilation (NNIV) than during spontaneous breathing (SB) in a neuromuscular population requiring nocturnal and daytime non-invasive ventilation.

Method:

Cross over, randomized, open study, the patients acting their own controls. Two situation were studied: SB vs. NNIV

During each condition, five swallowing of 2 different of volumes of water (5 or 10 ml) followed by 5 teaspoon (5ml) of textured yogurt (Danette, Danone).

Evaluation included duration of swallowing, number of swallow per bolus, number of respiratory cycles required per bolus and percentage of swallows followed by expiration. Dyspnea during swallowing was also evaluated.

Selection criteria

Adult patients, followed for neuromuscular disorders, requiring nocturnal and daytime non-invasive ventilation, clinically stable at the time of the study.

Number of patients, center

Ten subjects will be included in the study. Subjects will be recruited during the home ventilation unit of the intensive care department of the Raymond Poincaré hospital during the hospitalization for the usual follow-up of their respiratory failure.

Duration of study: 24 months.

2 SCIENTIFIC JUSTIFICATION AND GENERAL DESCRIPTION GÉNÉRALE OF RESEARCH

2.1 General description of device

2.1.1 Ventilator

Modified Elisee 150 (ResMed) (description below).

2.1.2 Switch

We recently conducted a clinical research study using a ventilator which had one parameter that could be automatically modified by a switch controlled by a neuromuscular patient. The aim was to optimize speech of patient by providing ventilator settings appropriate for speech (but not for

rest) and therefore, as soon as the patient wanted to speak, he could use the switch. Using the same principle of patient's control of the ventilator, we want that, as soon as the patient decides to swallow, using the same type of switch, he is able to stop the ventilator from generating an insufflation while he is swallowing. This option given to the patient provides additional security. He can decide to stop ventilation whenever he wants to, without taking off the ventilation interface, while previously he would have needed to adapt his swallowing to the ventilator rhythm which limited the use of ventilation while feeding and usually imposed to feed while spontaneously breathing.

2.2 Summary of previous available and relevant non-clinical and clinical studies

See « context » chapter. Outside of our previous published work, we have observed clinically patients that eat while they are mechanically ventilated with a nasal mask and who manage to swallow during the expiratory time set on the ventilator. The rehabilitation team of Inkendaal (Inkendaalstraat 1, B-1602 Vlezenbeek, Brussels, Belgium) also shared with us their experience of patients who use a mechanical hyperinflation through a mouth piece right before putting food in their mouth and swallowing. We think that it would be simpler for the patients to take the food bolus in their mouth if they are ventilated with a nasal mask which would allow them to masticate if necessary and then to stop ventilation when they decide to swallow.

2.3 Summary of expected benefits and risks

We expect swallowing to be faster, less fragmented, better synchronized with ventilation and that dyspnea will decreased while under NNIV compared to SB.

As the device used is under the patient's control through a switch, we do not expect side effect. Indeed, the patient may resume mechanical ventilation whenever he wants by releasing the switch.

2.4 Description of study population.

The patients studied will be adult patient followed for a neuromuscular chronic respiratory failure requiring non-invasive ventilation. They will usually be ventilated during daytime and nighttime through a nasal interface or a mouthpiece for a minimum duration of 14h/day.

Patients will be clinically stable at the time of the study, without any recent episode of acute respiratory failure.

3 RESEARCH OBJECTIVES.

3.1 Main objective

To demonstrate that an adapted nasal ventilation improves swallowing parameters (duration, number of swallows, number of respiratory cycles per bolys) of neuromuscular patients with respiratory failure.

3.2 Secondary objective(s)

To demonstrate that an adapted nasal ventilation improves ventilation-swallowing synchronization and decreases dyspnea during swallowing of neuromuscular patients with respiratory failure.

4 RESEARCH CONCEPT.

4.1 Primary study endpoint

Swallowing efficiency evaluated by three criteria:

- Swallowing duration
- Number of swallows per bolus
- Number of respiratory cycles per bolus

4.2 Secondary Study Endpoint(s)

- Percentage of swallows followed by an expiration
- Dyspnea sensation evaluated with the Borg scale

4.3 Experimental plan – practical layout

This is a cross-over, randomized, open study, the patients being their own control.
Patients will be recruited during their usual respiratory follow-up in the home ventilation unit of the intensive care department of the Raymond Poincaré hospital. These follow-ups require 2 to 3 days of hospitalization in order to perform the necessary exploration to evaluate respiratory function and ventilation efficiency of these patients.
The patients will be contacted upon their arrival, the protocol will be proposed and explained by one of the physician investigator and the informed consent form will be given to them.
If the patients choose to participate to the study, it will be performed the following day before the patient's programmed discharge. Hospitalization duration should not be extended by the protocol. The protocol does not entail any change of patient's treatment or management; therefore no control visit is planned.

Trial procedure

The evaluation related to the study will last about 1 hour 30. It will be performed in the intensive care department, in the presence of the intensivist physicians, with an emergency cart available in case of adverse event.
A continuous transcutaneous recording of oxygen saturation and cardiac frequency will be performed throughout the protocol with an oxymeter (Ohmeda Biox, BOC Healthcare, Boulder, CO). The patients will be explorer comfortably seated, in a position similar to their usual position during meals. Tests will be started after a ten minutes rest periods, during which the different sensors are placed. Patient's head is positioned in neutral position in order not to influence swallowing parameters [6] and to ensure homogeneity of recordings. The head position will be recorded by measuring chin-sternum distance.
The ventilator used during the evaluation will be set with the usual ventilation parameters of the patients and adequacy of ventilation will be checked before starting the swallowing evaluation. The patient will also be familiarized with the switch. Swallowing exploration will involve five series of water boluses of 2 different volumes (5 and 10 ml) which will be administered in random order. The water boluses will be placed in the oropharynx with a 20ml syringe. Each patient will be recorded during spontaneous breathing without ventilatory support and then with nasal non-invasive ventilation. Each situation will be applied in random order. Subjects will not be informed of the water bolus volume. Instructions will be given that, once they are signaled, the patient should swallow as usual, trying to be as efficient as possible. After a rest period and in the absence of detectable aspiration with water boluses, the patients will be recorded while swallowing 5 successive teaspoon of textured yogurt (Danette, Danone®). In order not to be limited by the motor dysfunction, the yogurt will be administered by a third party. This recording will be done while ventilated and without any ventilator support.
Ventilatory situation will be done in randomized order.

Swallowing study

A non- invasive evaluation, validated in previous publications [7], initially performed to demonstrate swallowing impairment in muscular and neurological disorders [7-10], will be used.
The evaluation consists of the simultaneous recording of the following parameters:

- Electromyographic activity of the sus-hyoïdial muscles with skin-surface bipolar electrodes taped and placed under the chin next to the floor of the oral cavity. Signal was amplified and filtered (band width 45 to 55 Hz), straightened and integrated.
- Laryngeal motion with piezoelectric sensor taped with transparent tape (Tegaderm,3 M Health Care, St Paul, USA) on the midline between the cricoid and thyroid cartilages (about 2cm above the tracheostomy tube in the tracheostomized patients). Signal was amplified and filtered (band width 45 to 55 Hz).

Correlation of EMG and laryngeal signals will allow distinguishing during analysis between artefacts and real swallowing.

Respiration exploration during swallowing will be performed by collecting the following parameters:
Thoracic and abdominal respiratory motion are recorded with a inductive plethysmograph (Respitrace ; Ambulatory Monitoring, Ardsley, NY). Thoracic bands will be placed around the thorax above the nipples line; abdominal bands will be place on the umbilical line.
During recordings performed under non-invasive ventilation, pressure was recorded on the inspiratory circuit of the ventilator with a differential pressure captor (Valydine MP 45 ± 100 cm H_2O, California, USA). Likewise, flow delivered by the ventilator will be measured with by a pneumotachograph (Fleisch#1, Lausanne, Suisse) connected to a differential pressure captor (Valydine MP 45 ± 5 cm H_2O, California, USA)
Acquisition system:
All the respiratory and swallowing parameters will be recorded simultaneously by an acquisition system able to digitize analogical signals (MP150, Biopac System, Santa Barbara, CA) and then treated with the Acknowledge program (Biopac System). All the data will be recorded on a computer, via an analogical-digital system (MP150, Biopac System, Santa Barbara, CA).

4.4 Description of the steps taken to reduce and to avoid bias

In order to avoid a learning effect bias during the procedure, the order of the ventilation conditions (SB and NNIV) will be randomized.

4.5 Study duration

For each patient, the evaluation in each condition should not exceed 30 minute. Therefore, the total duration of the procedure should not exceed 1 hour 30 minuters.
Expected study duration, including patients' recruitment and inclusion as well as data analysis is 24 months.

4.6 Description of the rules for definitive or temporary study interruption

As the device used is under the patient's control through a switch, and as the study does not involve any invasive procedure, no severe adverse effect is expected. Should this not be the case, the expected conduct is to place the patient under his usual ventilator and to inform the on-call intensivist physician.

4.7 Identification of the data directly collected from the observation handbook and which will be considered as source data.

General data:
Inclusion date
Family name, first name (first letters)
Sex
Date of birth, age
Neuromuscular disorder diagnosis
Hospitalization date
Mean duration of meal (evaluated by the patient and his entourage / evaluated during hospitalization)
Bulbar Norris Scale
Mallampati score
Current treatment
Ventilator type/ model /version
Ventilation duration/day
Date of diurnal NIV
Ventilation parameters:
Inspiratory pressure
Respiratory rate
Minute ventilation or tidal volume
FiO2
Trigger
I:E

Clinical examination
Weight, height or arm-span, body mass index
Cardiac frequency
Oxygen saturation
Respiratory rate
Pulmonary function tests
FEV1
FVC
VC
FEV1/FVC
FEV1/VC
FRC
RV
TLC
Pi max
Pe max
SNIP
Arterial Blood gases
FiO_2 or O_2 prescription
PO_2
PCO_2
pH
In each situation (SB vs. NNIV)
Cardiac frequency
Oxygen saturation
Respiratory rate
Respiratory comfort (Borg)
Swallowing duration
Number of swallows per bolus
Number of respiratory cycles per bolus
% of swallows followed by expiration

5 SELECTION OF STUDY POPULATION

Patients will be recruited among neuromuscular patients requiring long-term home ventilation during nighttime and daytime, and followed for their respiratory management in the home ventilation unit of the Intensive care unit of the Raymond Poincaré hospital.

5.1 Inclusion Criteria

- Patients followed for a neuromuscular chronic restrictive respiratory failure regardless its etiology, without a suprabulbar involvement associated
- Adult man or woman ≥18 years old
- Planned hospitalization in the “home ventilation” unit of the Raymond Poincaré hospital.
- Extended ventilatory support with nighttime and daytime NIV for at least 14h/day.
- Remaining respiration autonomy of at least 1 hour per day (allowing meals)
- Stable clinical state at the time of the study
- Completion of a prerequisite medical examination
- Signed patient consent form
- Negative pregnancy test for women of reproductive age or efficient contraception.

5.2 Exclusion Criteria

- Patient’s refusal to participate to the study
- Inability to cooperate
- Hemodynamic instability
- Acute respiratory failure

- Non inscription to social security (Beneficiary or entitled person)
- Patient under guardianship or trusteeship

5.3 Rules concerning simultaneous participation to another research study, exclusion period

No exclusion period preventing the patient to participate to other biomedical research is planned.

6 PREMATURE STOP OF DEVICE(S) USE

In case of patient's request or of unexpected adverse effect

7 TREATMENTS USED OUTSIDE OF THE DEVICE CONCERNED BY THE STUDY

No modification of the patients' usual treatment is planned during the protocol. No other associated treatment is planned with the protocol. Therefore, no specific follow-up is planned at the end of the protocol.

8 PERFORMANCE EVALUATION

Performance will be evaluated according to the following parameters:

Swallowing efficiency

- Swallowing duration
- Number of swallows per bolus
- Number of respiratory cycles per bolus
- % of swallows followed by an expiration
- Swallowing comfort (AVS)

Tolerance

The following respiratory parameters will be collected before and after each swallowing in the different conditions:

- Cardiac frequency
- Oxygen saturation
- Respiratory rate
- Respiratory comfort (Borg)

Switch use

- In each situation (duration of switch use/duration of swallowing)
- In each situation: (number of switch activation/time unit)
- Usefulness of switch (AVS)
- Interest of the device (yes/no/no opinion)

9 SECURITY EVALUATION

9.1 Description of parameters for security evaluation

As the usual ventilation parameters of the patients are not modified, no severe adverse effect is expected.

In case of poor tolerance of NNIV, the patient will be switched to his usual ventilator with its usual parameters.

The totality of the evaluation will be performed in presence of a physician familiar with ventilation techniques.

Respiratory tolerance of the evaluation will be monitored by a continuous recording of percutaneous oxygen saturation, cardiac frequency and respiratory rate.

Adverse event

Any harmful manifestation occurring to the person participating to a biomedical research whether or not this harmful manifestation is related to any experimental part of the research, regardless if it concerns performed actions or tested products.

Adverse effect

Any harmful and unwanted reaction at any experimental part of the research, regardless if it concerns performed actions or tested products.

Severe adverse event or effect
Any adverse manifestation or reaction leading to the death, endangering the life of the person participating to a biomedical research, requiring an hospitalization or the extension of an hospitalization, leading to important and lasting disability or deficiency, or leading to a congenital anomaly or malformation.

Unexpected adverse effect
Any adverse effect which nature, severity or evolution is not in accordance with the informations featured in the official reference lists of the authorities.

New fact
Any security information, liable to lead to a reevaluation of the benefits and risks balance of the research or that could be sufficient to lead to modification of the documents related to the research, to the management of the research and, eventually, to the use of the product.

9.2 Methods and calendar for measurement, collection and analysis of parameters for security evaluation

Pilot committee
It will include clinicians initiating the project, the biostatistician in charge of the project, representative of the promoter and of the CIC-IT.
It will define the general organization and progress of the research and will coordinate the informations. It will initially determine methodology and will decide during the research conduction of the steps to be taken in case of unexpected events, will monitor the study progress especially concerning tolerance and adverse events.

9.3 Procedures adopted for recording and reporting adverse events

9.3.1 Not severe adverse event:
Any adverse event –not severe as defined previously- observed during the research and consequently will be report on the observation handbook in the required field.
Only one event should be reported per field. The event can be a symptom, a diagnosis or the result of complementary exam considered significant. All the clinical and paraclinical elements allowing a better description of the event should be reported.

9.3.2 Severe adverse event (SAE):
The notification form for severe adverse event, validated for research, is included in the annex of the protocol.
The investigator should **immediately** notify the promoter of all the severe adverse events.
The investigator completes the notification form of severe adverse event (from the research observation handbook) and sends it to the promoter by fax to 01 46 97 16 97 within 48 hours
The investigator also has to inform the CIC-IT in charge of the research of the occurrence of a SAE.
For each severe adverse event, **the investigator will have to issue an opinion on the causal connection between the event and any experimental part of the research, regardless if it concerns performed actions or tested products.**
Obtaining information on the description and the evaluation of the adverse event may not be possible within the time delay for initial notification.
Therefore, clinical evolution as well as the results of any eventual clinical check-up and diagnostic and/or laboratory exams, or any other information allowing an adequate analysis of the causal connection should be reported:

- **Either, on the initial notification form of the SAE, if they are immediately available.**

- **Either, later and as soon as possible, by sending by fax a new and updated SAE notification form (specifying that it is the follow-up of an already notified SAE and indicating the follow-up number).**

Any notification done by the investigators should identify the subject affected **by a unique code number** attributed to each research subject.

In case of notified death of a subject participating to a research**, the investigator will communicate to the promoter all the complementary information required** (hospitalization report, autopsy report…).

Any new fact occurring in the research or in the context of the research, from either the literature or other ongoing researches should be notified to the promoter.

- **Notification of severe adverse events to Health Authorities**

It will be ensured by the promoter, after evaluating the severity of the adverse event, its potential link to the experimental element of the research, regardless if it concerns performed actions or tested products and the unexpected nature of the adverse effects.

All suspicions of unexpected severe adverse will be notified by the promoter to the appropriate authorities within the legal delays.

Any safety data or new fact that could significantly modify the evaluation of the risks and benefits balance of the experimental element of the research (regardless if it concerns performed actions or tested products) or of the research itself, or which could lead to consider modifications regarding the conduct of research, will be notified by the promoter to the competent authorities, to the Study Participant Protection Committee, and to the research investigators.

For example:

a) Any clinically significant increase in the frequency of occurrence of an expected severe adverse effect.
b) Suspicions of an unexpected severe adverse effect in participants who have finished the study and which have been notified by the investigator to the promoter, as well as potential follow-up reports.
c) Any new fact related to the conduct of the clinical trial, when this new fact is liable to jeopardize participants' safety. For example:
- A severe adverse event liable to be linked to the investigations or to the diagnostic produre of the essay and that could modify the conduct of the clinical trial.
- A significant risk for the trial population as, for example, the lack of efficiency of the experimental element used in the treatment of a life-threatening disease.
- Significant safety results resulting for a recently finished study conducted on an animal model (such a study on carcinogenesis)
- The early stop of the temporary suspension for safety reason of trial conducted on the same experimental element in another country.

d) The recommendations of the independent surveillance committee [Data Monitoring Committee (DMC) ou Data Safety Monitoring Board (DSMB)], if need be, if they are relevant for the safety of participants.
e) Any unexpected severe adverse effect transmitted to the promoter by the other promoter of a clinical trial conducted in a different country on the same experimental element.

9.4. Procedures and follow-up of participants following adverse events

Any patient who presents an adverse event needs to be followed-up until its resolution or its stabilization.

If the event is not severe, the evolution will be noted in the corresponding page of the research observation handbook in the intended section.

If the event is severe, a follow-up of Severe Adverse Event will be sent to the promoter.

10 STATISTICS

10.1 Number of participants to be included in the study, and number of patients in each study center with the statistical justification.

Ten subjects will be included in the study. A previous study in the department on the same topic of research performed on 7 patients and comparing 2 types of ventilation showed highly significant differences [11]. Therefore, 10 patients should allow reaching similar significant differences. The patients will be recruited in the Home Ventilation Unit of the Intensive Care Department of the Raymond Poincaré Hospital during their usual respiratory follow-up.

10.2 Description of planned statistical analysis, including calendar of planned intermediary analysis

This is an efficiency study and the main analysis will be done in intention to treat all the randomized subjects according to the strategy resulting from randomization, independently of the received treatment and the protocol deviation. An exclusion criterion is the patient's consent withdrawal.

The data will be summarized according to group, with the usual descriptive statistics according to the nature of the variable:

- Quantitative date: n, minimum, maximum, median, mean, standard-deviation, range and interquartile range.
- Qualitative variable: n, population, percentages.

The statistical method used will be comparison of percentages, means and/or medians of differences from zero.

The quantitative criteria (results obtained with different methods to improve swallowing) will be compared by a two-way repeated measures analysis (NIV, SB), type of boluses swallowed (water 5ml, water 10 ml, textured yogurt Danette 5ml).

Tolerance analysis will be performed in intention to treat.

Adverse events will be described in terms of frequency and presented according to the treatment group, the type of event, the causal relationship (certain, probable, possible, unlinked) and their intensity.

P values below 0.05 will be considered significant.

Statistical analysis will be performed with the R and S-plus software of the CIC-IT

10.3 Methods for missing, unused or non-valid data management

It will be monitored whether the rate of missing or non-valid data is similar in the different treatment group. However, if a difference is observed, the causality will be investigated.

The inclusion of the missing data on the main criteria will be performed according to the situations' complexity (replacement with the last available data, replacement by the mean effect of the group…)

A sensibility analysis will be performed.

10.4 Intermediary analysis

No intermediary analysis is planned

10.5 Choice of persons to be included in the analysis

Patients' status: selected, randomized, evaluated in intention to treat, lost to follow-up or study interruption, protocol deviations, will be described and compared between groups.

11 RIGHT OF ACCESS TO DATA AND SOURCE DOCUMENTS

The persons with a direct access in accordance with the applicable laws and regulations in effect, in particular the L.1121-3 and R.5121-13 articles of the Public Health Code (for example, the investigators, the persons in charge of quality control, the monitors, the clinical research assistants, the auditors, and any person liable to collaborate to the trials) will take all necessary precautions in order to ensure the confidentiality of the informations relating to the experimental drugs, the trials, the persons participating and, in particular regarding to their identity and the results. Collected data by the persons during the quality control and the audits will be anonymised.

12 QUALITY CONTROL AND GUARANTEES

The research will be constrained by the promoter usual operating procedures. The conduct of the research in the investigative centers and the management of the subjects will be done in compliance with the Helsinki Declaration and the Good Practices rules in effect

12.1. Monitoring procedures

The clinical research assistants (CRA) of the promoter will visit the investigative centers with a rate in accordance with the follow-up planning of patients scheduled in the protocol, with the inclusions in the different centers and with the risk level attributed to the research.

- Opening visit in each center: prior to inclusion, for the introduction to the protocol and familiarization with the different participants to the biomedical research.
- During the following visits, the observation handbook will be prospectively reviewed according to the state of advancement of the research by the CRA. The principal investigator and the associated investigators who include or ensure the follow-up of the persons participating to the research will commit to meeting regularly with the CRA.

During these site visits and in compliance with the Good Clinical Practice rules, the following elements will be reviewed:

Compliance with the protocol and the procedures defined for the research,

Verification of the patients' signed informed consent forms,

Verification of the source documents and confrontation with the data collected in the observation handbook regarding accuracy, missing data.

- Closing visit: recovery of observation handbook, evaluation with the pharmacy, document of the biomedical research, document storage.

12.2 Data transcription in the observation handbook

All the information required for the protocol has to be collected in the observation handbook and the investigator must provide an explanation for each missing data.

The data will be transferred to the observation handbook as soon as they are available and whether they concern clinical or paraclinical data. Data should be copied in black ink in a clear and legible way (in order to facilitate reproduction and computer entry).

Incorrect data, identified in the observation handbooks, will be clearly crossed out and the nex data will be copied in the handbook with the initials and the date by the member of the investigative team who performed the correction.

Subjects' anonymity will be ensured by mentioning the first letter of the family name, the first letter of the first name and the inclusion number on all the documents required for the research.

The filed computerized data will be registered with the CNIL (the French National Commission for Information technology and Liberties) accordingly to the relevant procedure.

13 ETHICAL CONSIDERATIONS

The promoter is defined by the 2004-806 law of August 9th 2004. In this research, **ADEP ASSISTANCE is the promoter.**

Prior to research initiation, each investigator will to the research promoter's representative a signed and dated copy of his/her resume including his/her inscription number to the Medical Council and ADELI national number.

13.1 Authorization request to the competent authority

In order to start the research, the promoter must submit an authorization request file to the competent authority the Afssaps (French Health Products Safety Agency). The competent authority, defined by the article L.1123-12, rules on the safety of the persons participating to a biomedical research, taking account in particular of the safety and the quality used during the research in accordance with, when appropriate, the existing referential, their condition of use and the security of the persons regarding the acts performed and the methods used as well as the intended modalities for the participants' follow-up.

13.2 Request for opinion with the Study Participant Protection Committee (CPP)

In accordance with the L.1123-6 article of the Public Health Code, the research protocol must be submitted by the promoter to a Study Participant Protection Committee. The committee's opinion is notified to the competent authorities by the promoter before the research is initiated.
The promoter must be informed by the principal investigator of any modification project of the protocol.
The modifications will be qualified as substantial or nor. A substantial modification is a modification liable, in any manner, to modify the guarantees provided to the persons participating to the biomedical research (modification of inclusion criteria, extension of the inclusion duration, and participation of new centers…).
After the research is started, any substantial modification initiated by the promoter must obtain, prior to its application, a favorable opinion from the committee and the authorization from the competent authorities. In that case, if necessary, the committee will ensure that a new consent form for participants to the research is collected.
Furthermore, any research extension (profound modification of the therapeutic plan or of the included population, extension of the treatment duration and/or of the therapeutic acts not scheduled initially in the protocol) shall be considered as a new research.
Any substantial modification will lead the promoter, after tax payment, to submit an authorization request file to the Afssaps and/or to request an opinion from the CPP.

13.3 CNIL Notification

The law requires that the notification of the computerized file containing personal data collected for research should be made before the actual initiation of the research.
A reference methodology specific of the personal data processing performed within the field of biomedical researches defined by the 2004-806 law of August 9th 2004 as it pertains to the L.1121-1 and following articles of the Public Health Code was defined by the CNIL in January 2006.
This methodology allows a procedure of simplified notification when the nature of the data collected during the research is compatible with the list established by the CNIL in its reference documentation.
When the protocol includes a quality control of the data by CRA representing the promoter and when it falls within the scope of the CNIL simplified notification procedure, ADEP assistance as the study promoter will request from the manager of the computer file to agree in writing to respect the simplified reference methodology MR06001.

13.4 Information note and informed consent

No biomedical research can be undertaken on a person without his/her free and informed consent, collected after being given the information as specified in the L.1122-1.
Consent has to be given in writing or, in case of impossibility, attested by a third party. This third party has to be completely independent from the investigator of the promoter.

13.5 Final research report

The final research report will be written in collaboration between the main investigator and the biostatistician of the research. The report will be submitted to each investigator for his/her opinion. When a consensus is reached, the final version will be endorsed by the signature of each investigator and addressed to the promoter in the shortest delay after the effective ending of the research. A report written accordingly with the reference model of the competent should be transmitted to the reference authority and to the Study Participant Protection Committee within a year, after the end of research, defined as the last visit of the last included patient. This delay is shortened to 90 days in case of premature interruption of the research.

14 DATA TREATMENT AND STORAGE

The research documents falling in the scope of the law on biomedical research should be stored by all the concerned parties during a period of 15 years after the end of the study. This indexed storage should entail:

- the copies of the authorization mail from the Afssaps and the obligatory opinion of the CPP,

- the successive version of the protocol (identified by the number of version and the date of version),
- the mails of correspondence with the promoter,
- the signed consent forms under closed envelope (in the case of underage subjects, signed by the legal guardian)
- the observation notebook completed and validated for each validated subject,
- all the annexes specific of the study,
- the final report including the statistical analysis and the study quality control (copy transmitted to the promoter)
- the certificates of the possible audits performed during the research.

The data base used for the statistical analysis will also be stored under the analyst responsibility (paper ou electronic copy)

15 FUNDING AND INSURANCE

15.1 Insurance

ADEP Assistance is the promoter of this research. In accordance with the law on biomedical research, it contracted insurance with Biomedi Insure for the entire duration of the research, warranting its own civil responsibility as well as that of all the participating parties (physician or employee involved in performing the research)(1004-806 law, Art L.1121-10 of CSP).

ADEP Assistance reserves the right to stop the research at any time for medical or administrative reasons; in that case, a notification will be provided to the investigator.

15.2 Scientific commitment

Each investigator will commit to respect the law obligations and to proceed through the research following to the Rules of Clinical Good Practice, in accordance with the Helsinki Declaration. To this end, a copy of the scientific commitment dated and signed by the main investigator of each clinical participating center will be given to the promoter.

16 RULES OF PUBLICATION

The promoter owns the data and no use or transmission to a third party can be done without the promoter's consent.

The first authors of the publication will be the persons who truly participated to the elaboration and the execution of the research and the drafting of the results.

ADEP Assistance should be mentioned as the promoter of the biomedical research and, eventually, as financial support.

17 BIBLIOGRAPHY

1. Willig TN, Paulus J, Lacau Saint Guily J, Beon C, Navarro J: **Swallowing problems in neuromuscular disorders**. *Arch Phys Med Rehabil* 1994, **75**(11):1175-1181.

2. Terzi N, Orlikowski D, Aegerter P, Lejaille M, Ruquet M, Zalcman G, Fermanian C, Raphael JC, Lofaso F: **Breathing-swallowing interaction in neuromuscular patients: a physiological evaluation**. *Am J Respir Crit Care Med* 2007, **175**(3):269-276.

3. Terzi N, Prigent H, Lejaille M, Falaize L, Annane D, Orlikowski D, Lofaso F: **Impact of tracheostomy on swallowing performance in Duchenne muscular dystrophy**. *Neuromuscul Disord* 2010, **20**(8):493-498.

4. Bonanno PC: **Swallowing dysfunction after tracheostomy**. *Ann Surg* 1971, **174**(1):29-33.
5. Terzi N: **Evaluation des troubles de la déglutition et des interactions ventilation/déglutition en cas d'insuffisance respiratoire chronique respiratoire d'origine neuromusculaire**. *Thèse de Sciences de la Vie et de la Santé.* Versailles: Université de Versailles Saint-Quentin-en-Yvelines; 2010.
6. Ertekin C, Keskin A, Kiylioglu N, Kirazli Y, On AY, Tarlaci S, Aydogdu I: **The effect of head and neck positions on oropharyngeal swallowing: a clinical and electrophysiologic study**. *Arch Phys Med Rehabil* 2001, **82**(9):1255-1260.
7. Ertekin C, Yuceyar N, Aydogdu I: **Clinical and electrophysiological evaluation of dysphagia in myasthenia gravis**. *J Neurol Neurosurg Psychiatry* 1998, **65**(6):848-856.
8. Ertekin C, Aydogdu I, Yuceyar N, Kiylioglu N, Tarlaci S, Uludag B: **Pathophysiological mechanisms of oropharyngeal dysphagia in amyotrophic lateral sclerosis**. *Brain* 2000, **123 (Pt 1)**:125-140.
9. Ertekin C, Tarlaci S, Aydogdu I, Kiylioglu N, Yuceyar N, Turman AB, Secil Y, Esmeli F: **Electrophysiological evaluation of pharyngeal phase of swallowing in patients with Parkinson's disease**. *Mov Disord* 2002, **17**(5):942-949.
10. Ertekin C, Yuceyar N, Aydogdu, Karasoy H: **Electrophysiological evaluation of oropharyngeal swallowing in myotonic dystrophy**. *J Neurol Neurosurg Psychiatry* 2001, **70**(3):363-371.
11. Terzi N, Prigent H, Lejaille M, Falaize L, Annane D, Orlikowski D, Lofaso F: **Impact of tracheostomy on swallowing performance in Duchenne muscular dystrophy**. *Neuromuscul Disord*.
